# Single-cell transcriptomic analysis reveals disparate effector differentiation pathways in human T_reg_ compartment

**DOI:** 10.1038/s41467-021-24213-6

**Published:** 2021-06-23

**Authors:** Yuechen Luo, Changlu Xu, Bing Wang, Qing Niu, Xiuhua Su, Yingnan Bai, Shuxian Zhu, Chunxiao Zhao, Yunyan Sun, Jiali Wang, Maolan Liu, Xiaolei Sun, Ge Song, Haidong Cui, Xiaoli Chen, Huifang Huang, Haikun Wang, Mingzhe Han, Erlie Jiang, Lihong Shi, Xiaoming Feng

**Affiliations:** 1 State Key Laboratory of Experimental Hematology, National Clinical Research Center for Blood Diseases, Institute of Hematology & Blood Diseases Hospital, Chinese Academy of Medical Sciences & Peking Union Medical College, Tianjin, China; 2 Novogene Co., Ltd., Beijing, China; 3 Hangzhou First People’s Hospital, Hangzhou, China; 4 Ganzhou Key Laboratory of Molecular Medicine, the Affiliated Ganzhou Hospital of Nanchang University, Ganzhou, China; 5 Central Laboratory, Fujian Medical University Union Hospital, Fuzhou, China; 6 CAS Key Laboratory of Molecular Virology and Immunology, Institut Pasteur of Shanghai, Chinese Academy of Sciences, Shanghai, China

**Keywords:** RNA sequencing, Mechanisms of disease, Regulatory T cells, Graft-versus-host disease, Stem-cell research

## Abstract

Human FOXP3^+^ regulatory T (T_reg_) cells are central to immune tolerance. However, their heterogeneity and differentiation remain incompletely understood. Here we use single-cell RNA and T cell receptor sequencing to resolve T_reg_ cells from healthy individuals and patients with or without acute graft-versus-host disease (aGVHD) who undergo stem cell transplantation. These analyses, combined with functional assays, separate T_reg_ cells into naïve, activated, and effector stages, and resolve the *HLA-DR*^hi^, *LIMS1*^hi^, highly suppressive *FOXP3*^hi^, and highly proliferative *MKI67*^hi^ effector subsets. Trajectory analysis assembles T_reg_ subsets into two differentiation paths (I/II) with distinctive phenotypic and functional programs, ending with the *FOXP3*^hi^ and *MKI67*^hi^ subsets, respectively. Transcription factors FOXP3 and SUB1 contribute to some Path I and Path II phenotypes, respectively. These *FOXP3*^hi^ and *MKI67*^hi^ subsets and two differentiation pathways are conserved in transplanted patients, despite having functional and migratory impairments under aGVHD. These findings expand the understanding of T_reg_ cell heterogeneity and differentiation and provide a single-cell atlas for the dissection of T_reg_ complexity in health and disease.

## Abstract (web summary)

Human Treg cells are central to immune tolerance, yet their heterogeneity and differentiation remain incompletely understood. Here the authors perform single-cell RNA and T cell receptor sequencing to resolve Treg cells from healthy individuals and patients with or without acute graft-versus-host disease revealing Treg complexity in health and disease.

## Introduction

Regulatory T (T_reg_) cells are a highly immunosuppressive population of CD4^+^ T cells characterized by the expression of the transcription factor forkhead box protein P3 (FOXP3). T_reg_ cells control immune responses and maintain peripheral tolerance^1–4^. T_reg_ cells have been suggested to be heterogeneous. Based on cell origins, T_reg_ cells are divided into two parts. Thymus-derived T_reg_ (tT_reg_) cells develop in the thymus and make up the majority of T_reg_ cell pool in secondary lymphoid organs. Peripheral T_reg_ (pT_reg_) cells arise from conventional T (T_con_) cells at peripheral inflammation sites with the acquisition of FOXP3 expression^5^. Human peripheral blood (PB) CD4^+^FOXP3^+^ T_reg_ cells are subdivided into three subpopulations: CD45RA^+^FOXP3^lo^/CD25^lo^ resting or naïve T_reg_ cells, CD45RA^−^FOXP3^hi^/CD25^hi^ effector T_reg_ cells, and CD45RA^−^FOXP3^lo^/CD25^lo^ cells (not true T_reg_ cells)^6^. Mass cytometry analysis based on 26 well-recognized T_reg_-associated markers identified 22 subsets^7^. T_reg_ cells can also be divided into several T helper (Th) like subpopulations^8^. However, none of these taxonomies are dependent on the unsupervised global gene expression profile.

Recent advances in single-cell RNA sequencing (scRNA-seq) have shed new light on T cell and T_reg_ cell heterogeneity at single-cell resolution^9–13^. Based on scRNA-seq, T_reg_ cells were subdivided into six clusters in healthy human PB^12^, or five clusters in the human breast cancer microenvironment^10^. The proportions of resting and activated T_reg_ populations in mice were reportedly not determined by T cell receptor (TCR) signaling strength, whereas the intensity of TCR signal intriguingly influenced the phenotypic and functional programs of activated T_reg_ cells^12^. T_reg_ cells also exhibited transcriptional dynamics along a continuum of tissue adaptation and presented conserved expression programs between homeostasis and disease and between mice and human^13^. Despite these findings, in-depth single-cell investigations on human T_reg_ cells during steady state or disease conditions are still limited. The identity, functional/homeostatic characteristics, differentiation, and relationships of distinct T_reg_ subsets remain incompletely understood.

T_reg_ cells are impaired in number or function in inflammatory disorders. Typically, graft-versus-host disease (GVHD) is a major adverse effect of allogeneic hematopoietic stem cell transplantation (allo-HSCT)^14^. GVHD is associated with the decreased number and function of T_reg_ cells^15,16^. Transfer of T_reg_ cells has alleviated GVHD symptoms in mouse models and clinical trials^17–19^. However, the mechanisms underlying T_reg_ cell defects in GVHD have not been fully addressed, at least partially due to the lack of single-cell omics analysis.

In this work, we use scRNA-seq and single-cell TCR (scTCR)-seq to analyze T_reg_ cells in PB and bone marrow (BM) from healthy donors and allo-HSCT patients with or without acute GVHD (aGVHD). Heterogeneous T_reg_ cell subpopulations are resolved. Their transcriptional signatures, phenotypic markers, and functional and homeostasis programs are defined. In addition, two T_reg_ cell differentiation pathways are identified, and their characteristics and transcription factors are defined. Moreover, T_reg_ cells from allo-HSCT patients with or without aGVHD are also analyzed at the single-cell level, which provide further insight into the conservation and change of T_reg_ cell dynamics under disease conditions.

## Results

### scRNA-seq resolves distinctive subsets among human *FOXP3*^+^ T_reg_ cells

**Fig. 1:**
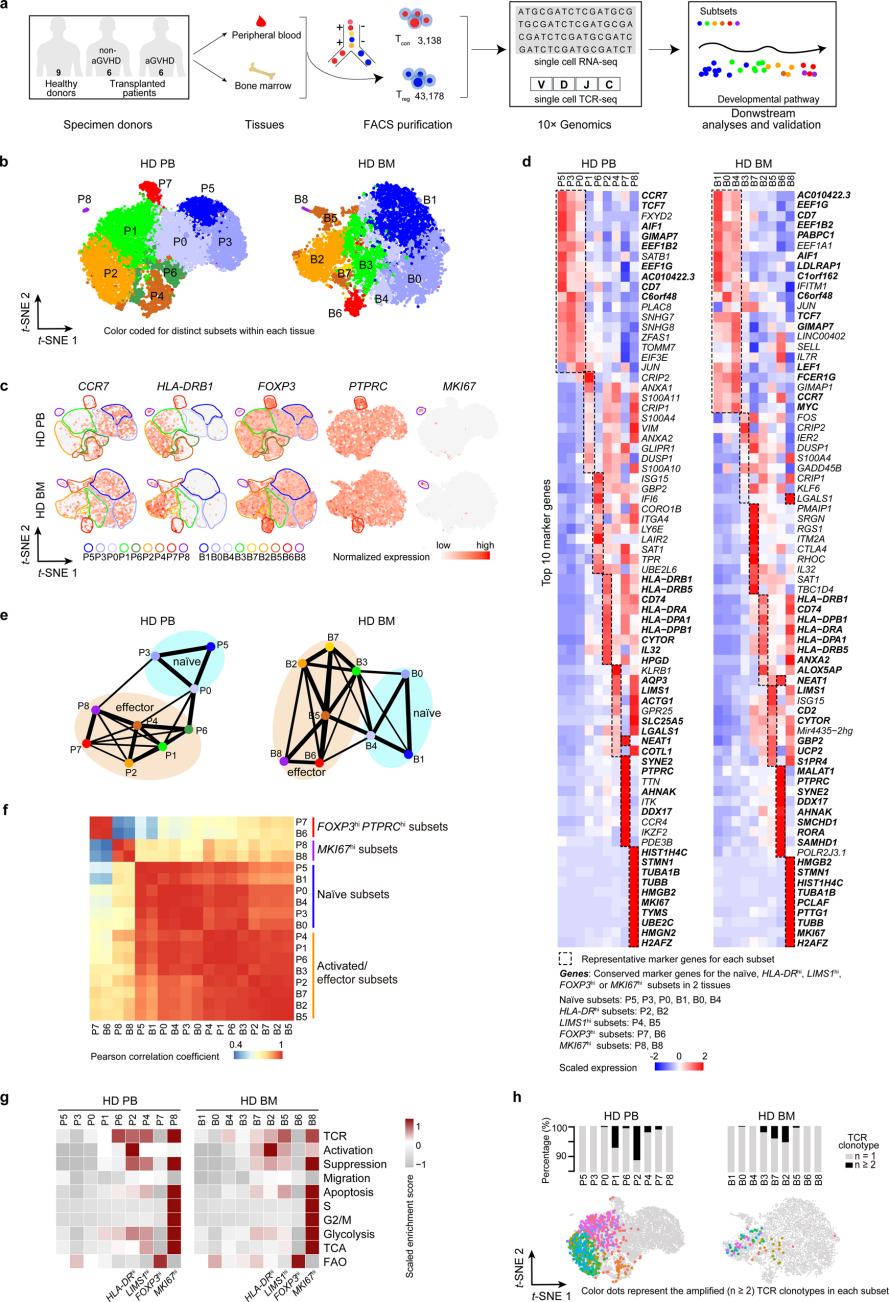
ScRNA-seq and TCR-seq reveals distinctive subsets among human *FOXP3*^+^ T_reg_ cells. **a** A scheme showing the overall strategy of this study. **b** *t*-SNE of single-cell transcriptomes of T_reg_ cells from healthy donor (HD) peripheral blood (PB, *n* = 8) and bone marrow (BM, *n* = 6) samples, colored by subsets. Each subset was numbered and labeled with the first letter (s) of the tissue name (P0 for cluster 0 in peripheral blood, etc.). **c** Projection of *CCR7*, *HLA-DRB1*, *FOXP3*, *PTPRC* and *MKI67* expression onto the *t*-SNE plot. **d** Heatmaps showing the top 10 (by fold change) marker genes for each subset, excluding the ribosomal and mitochondrial genes. Scaled expression means the gene expression was centered and scaled among subsets. The fold change means the values of normalized expression of genes in a specific subset compared to the normalized expression of genes in the other subsets. **e** Partition graph abstraction (PAGA) analysis. Nodes represent subsets, and thicker edges indicate stronger connectedness between subsets. **f** Correlograms visualizing the correlation of single-cell gene expression profiles between subsets from HD PB and HD BM. **g** Heatmaps showing the GSVA enrichment score of T_reg_ cell feature pathways for each subset. **h** The amplified (*n* ≥ 2) TCR distribution of T_reg_ cells across different subsets, colored by TCR clonotypes. The insert pictures show the composition of unique and amplified (*n* ≥ 2) T_reg_ cell TCR clonotypes in each subset. Source data are provided as a Source Data file.

We firstly conducted scRNA-seq on T_reg_ cells from healthy donors and allo-HSCT patients with or without aGVHD. scRNA-seq was performed using the 10× Genomics Chromium platform to analyze CD4^+^CD25^+^CD127^−^ T_reg_^20,21^ and CD4^+^CD25^−^ conventional T (T_con_) cells sorted from PB and BM of healthy donors and allo-HSCT patients with or without aGVHD (Fig. 1a, Supplementary Fig. 1a–c, Supplementary Data 1). Consistent with a previous study^12^, not all CD4^+^CD25^+^CD127^−^ cells had *FOXP3* reads due to potential non-T_reg_ cell contamination and limited gene coverage in scRNA-seq (Supplementary Fig. 1d). To reduce the contamination of non-T_reg_ cell populations, *FOXP3*^*+*^ cells were selected for subsequent analyses. The 43,178 *FOXP3*^+^ T_reg_ cells and 3,138 *CD4*^+^*FOXP3*^−^ T_con_ cells were analyzed and they had an average of 1331 genes per cell (Supplementary Fig. 1e, f, Supplementary Data 2). The T_reg_ identity of these *FOXP3*^+^ cells was confirmed by their enriched expression of canonical T_reg_ marker genes including *FOXP3*, *IKZF*2, *TIGIT*, *IL2RA*, *IL10RA* and *CTLA4* (Supplementary Fig. 1g, Supplementary Data 3)^3,4^.

The heterogeneity of healthy donor T_reg_ cells was assessed. Cell clusters were identified based on the shared nearest neighbor (SNN) clustering algorithm in Seurat^22^ and visualized through *t*-distributed stochastic neighbor embedding (*t*-SNE) or Uniform Manifold Approximation and Projection (UMAP) analysis. Finally, nine clusters in PB and BM T_reg_ cells from healthy donors were defined (Fig. 1b, Supplementary Fig. 2a). According to previously defined markers of naïve and effector T_reg_ cells, including CCR7, HLA-DR, and FOXP3^23–25^, and the top 10 signature genes and signature transcription factors of each cluster (Fig. 1c, d, Supplementary Fig. 2b–f, Supplementary Data 4), clusters 0, 3, and 5 in PB (P0, P3, P5) and clusters 0, 1, and 4 in BM (B0, B1, B4) perfectly matched naïve status with *CCR7*^hi^*TCF7*^hi^*HLA-DR*^low^*FOXP3*^low^ profile. These clusters were termed naïve subsets. The others were activated/effector subsets. They featured negative or low expression of *CCR7*. In addition, the partition-based graph abstraction (PAGA) mapping revealed high connectivity among the naïve clusters, and among the activated/effector clusters (Fig. 1e). To better resolve these activated/ effector subsets, we performed a further annotation. Among them, clusters 2 in PB and BM (P2, B2) exhibited high expression of *HLA-DR* and were named as *HLA-DR*^hi^ subsets. The expression of *LIMS1* was high in clusters 4 and 8 in PB (P4, P8) and clusters 5 and 8 in BM (B5, B8). Among these clusters, P4 and B5 were designated *LIMS1*^hi^ subsets. Cluster 7 in PB (P7) and cluster 6 in BM (B6) were highly similar in transcriptome signature and had the *FOXP3*^hi^*PTPRC*^hi^*DDX17*^hi^ profile. P7 and B6 were designated *FOXP3*^hi^ effector subsets. Cluster 8 in PB (P8) and cluster 8 in BM (B8) were very similar, both of them had an *MKI67*^hi^ *TUBB*^hi^*HMGB2*^hi^ profile and the maximal proportion of cells in the S + G2/M phase (Supplementary Fig. 3a). P8 and B8 were designated *MKI67*^hi^ effector subsets. Although the *FOXP3*^hi^ and *MKI67*^hi^ T_reg_ cell subsets were highly conserved across PB and BM, they also showed tissue-specific gene expression differences (Fig. 1f, Supplementary Fig. 3b, Supplementary Data 5). These differences might have been caused by the distinctive tissue microenvironment. T_reg_ subsets also displayed disparate expression of signature transcription factors (Supplementary Fig. 2f). There were more naïve T_reg_ cells in BM than those in PB, although there was no significant difference (Supplementary Fig. 3c). The naïve and effector T_reg_ cell markers suggested by T_reg_ cell scRNA-seq studies^12,13^ were also enriched in our defined naïve and activated/effector subsets, respectively (Supplementary Fig. 3d, e). Thus, based on unsupervised clustering, we identified several distinct human T_reg_ cell subsets under steady-state conditions.

To compare the functional/homeostatic features among different clusters, gene set variation analysis (GSVA)^26,27^ was performed (Fig. 1g, Supplementary Data 6). GSVA showed a gradual increase in TCR signaling, activation, and suppressive function from naïve to activated/effector subsets. The *MKI67*^hi^ subsets had the highest expression of TCR signal, suppression, apoptosis, cell cycle (S, G2/M), glycolysis, and tricarboxylic acid cycle (TCA) gene sets. The *FOXP3*^hi^ subsets were characterized by low TCR signal and glycolysis, intermediate suppression, and high fatty acid oxidation (FAO) gene sets. The *HLA-DR*^hi^ subsets exhibited the highest degree of activation. Further examination of individual genes indicated that suppression and migration genes were selectively expressed by different subsets (Supplementary Fig. 3f). Particularly, the *HLA-DR*^hi^ subset strongly expressed *TGFB1*, *CCR3* and *CCR10*. The *LIMS1*^hi^ subset highly expressed *IL10* and *CCR9*. The *FOXP3*^hi^ subset highly expressed *IL2RA*, *CTLA4*, *CCR4*, *CXCR4* and *SELL*. The *MKI67*^hi^ subset highly expressed *LGALS1* and *ITGAE*.

### scTCR-seq reveals amplification and transition among T_reg_ cells subsets

TCRs are uniquely expressed by individual T_reg_ cell clones^28,29^. To trace the amplification history of a single T_reg_ clone, scRNA-seq and paired scTCR-seq were performed simultaneously in 11,830 sorted *FOXP3*^+^ T_reg_ cells and 1,519 sorted T_con_ cells (Supplementary Data 7). This approach allowed direct mapping of gene expression to TCR in the same cell. Most amplified TCR clonotypes (*n* ≥ 2) were detected in activated/effector subsets such as *HLA-DR*^hi^ and *LIMS1*^hi^ effector subsets. No or very few cells from naïve, *FOXP3*^hi^, or *MKI67*^hi^ subsets harbored clonally amplified TCRs (Fig. 1h). We also identified a total of 250 and 27 TCR clonotypes that were shared by cells from at least two different clusters in PB and BM, respectively (Supplementary Fig. 3g). Most TCRs were shared between activated/effector subsets, suggesting frequent differentiation between them. Although either *FOXP3*^hi^ subset or *MKI67*^hi^ subset had shared TCR clonotypes with other subsets, there was no shared clonotype between *FOXP3*^hi^ subset and *MKI67*^hi^ subset, which might be due to their distinctive development or the limited cell number. The latter would not be sufficient to capture the same TCR (many TCR clonotypes are present in vivo). In addition, 121 TCR clonotypes were shared between PB and BM T_reg_ cells (Supplementary Fig. 3h). The TCRs shared between tissues were presented in most subsets, except P5 (naïve), P3 (naïve), P8 (*MKI67*^hi^), and B8 (*MKI67*^hi^). Therefore, P5 and P3 may be at the most naïve status that were only present in PB. *MKI67*^hi^ subsets might differentiate independently in PB or BM. However, the lack of shared TCRs between PB and BM *MKI67*^hi^ subsets may have been caused by a relatively small cell number of analyzed cells. These results collectively indicated a process of naïve to activated and effector differentiation, defined as the transition between individual T_reg_ subsets in one tissue or between two tissues. However, we could not determine the order of T_reg_ cell development between different tissues. Further studies are necessary.

### In vitro assay of T_reg_ subsets

**Fig. 2:**
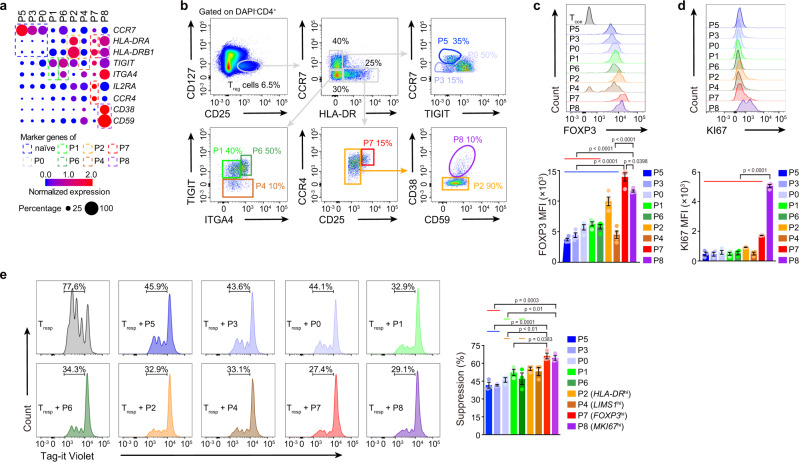
In vitro assay of T_reg_ cell subsets. **a** Dot plots showing the expression of indicated genes in each subset. **b** Gating strategy for HD PB T_reg_ cell subset sorting. **c**, **d** Flow cytometry and its quantification of FOXP3 (*n* = 4) and KI67 (*n* = 3) in each HD PB T_reg_ cell subset from PBMC. MFI, mean fluorescence intensity. In **c**, blue line indicated that P7 was compared with P5, P3, P0, P1, P6, P2 or P4; red line indicated that P8 was compared with P5, P3, P0, P1 or P6. In **d**, red line indicated that P8 was compared with P5, P3, P0, P1, P6, P2, P4 or P7. **e** In vitro suppression assay. Tag-it Violet-labeled T_resp_ cells were co-culture with different T_reg_ subsets for 96 h, with CD3/CD28 T cell activator (*n* = 3). The T_reg_: T_resp_ ratio was 1: 2. The Tag-it Violet dilution of T_resp_ cells was assessed by flow cytometry. Blue line indicated that P7 was compared with P5 or P3; orange line indicated that P7 was compared with P0 or P6; red line indicated that P8 was compared with P5 or P3; green line indicated that P8 was compared with P0 or P6. In **c**-**e**, experiment repeated at least three times; p values were determined by One-way ANOVA, part of the statistical significance was shown in the pictures and the whole ANOVA results were given in Supplementary Data 11; data are presented as mean values ± SEM. Source data are provided as a Source Data file.

To evaluate the functional and homeostatic characteristics of T_reg_ subsets in vitro, cell surface marker candidates for different clusters were identified (Fig. 2a), and clusters from PB were isolated by flow cytometry sorting (Fig. 2b, Supplementary Figs. 4a, 12a, f). Cells in the *FOXP3*^hi^ and *MKI67*^hi^ subsets were the largest and displayed the highest expression of FOXP3 and KI67 proteins, respectively (Fig. 2c, d, Supplementary Figs. 4b, 12a). The *FOXP3*^hi^ and *MKI67*^hi^ subsets exhibited the strongest inhibition of responder T (T_resp_) cell proliferation, followed by *LIMS1*^hi^ and *HLA-DR*^hi^ subsets and then by other activated/effector subsets (Fig. 2e, Supplementary Fig. 12a, b). *MKI67*^hi^ and *LIMS1*^hi^ subsets proliferated more than other activated/effector subsets after stimulation (Supplementary Figs. 4c, 12a, c). Activated/effector subsets had a slightly higher apoptosis rate than most naïve subsets during culture (Supplementary Figs. 4d, 12a, c). Surprisingly, a large proportion of cultured *LIMS1*^hi^ subset cells lost the FOXP3 protein (Supplementary Figs. 4e, 12a, c). The *LIMS1*^hi^ subset rarely expressed Helios (encoded by *IKZF2*) (Supplementary Figs. 4f, 12a), which is considered a potential marker of thymus-derived T_reg_ cells^30^. Although, the *LIMS1*^hi^ subset shared a number of TCRs with activated/effector T_reg_ subsets but not with T_con_ cells (Supplementary Fig. 4g), it still could not determine whether *LIMS1*^hi^ subset were converted from T_con_ cells or not. The collective in vitro findings further defined the functional and homeostatic characteristics of T_reg_ subsets previously resolved with single-cell transcriptomics.

### Two effector differentiation paths revealed by pseudotime analysis

**Fig. 3:**
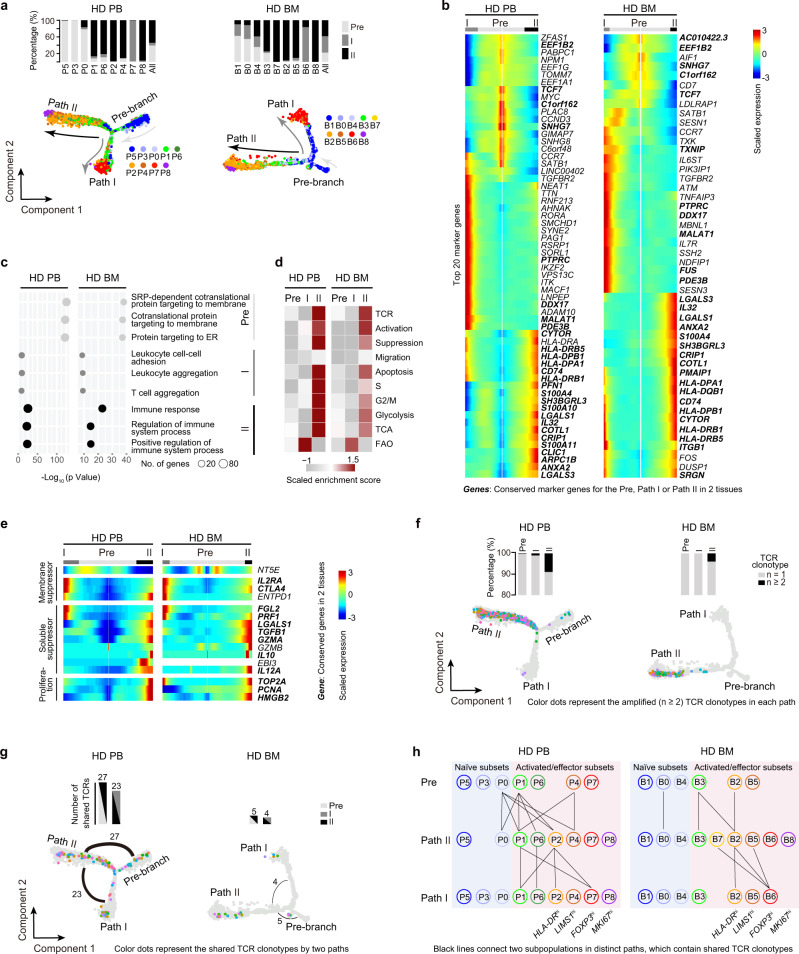
Pseudotime analysis and scTCR analysis defines two differentiation paths. **a** Pseudotime trajectory of T_reg_ cells within distinct tissues, colored by subsets. The insert picture shows the proportions of different T_reg_ cell paths in each cell subset (HD PB: *n* = 5, HD BM: *n* = 3). **b** Pseudotemporal gene-expression profiles of the top 20 (by fold change) marker genes for each path, excluding the ribosomal and mitochondrial genes. **c** Representative terms from Gene Ontology enrichment analysis of the differentially expressed genes for each T_reg_ cell path. **d** Heatmaps showing the GSVA enrichment score of T_reg_ cell feature pathways for each different path. **e** Pseudotemporal gene-expression profiles of suppression and proliferation-associated genes in the T_reg_ cell paths. **f** The amplified TCR distribution of T_reg_ cells across different paths, colored by TCR clonotypes. The insert pictures show the composition of unique and amplified (*n* ≥ 2) T_reg_ cell TCR clonotypes in each path. **g** The shared TCR distribution of T_reg_ cells between any two paths, colored by TCR clonotypes. The thickness of the black solid lines representing the relative numbers of shared TCRs. The insert pictures show the numbers of TCRs shared by two paths. **h** The shared TCR distribution of T_reg_ cells between any two subpopulations in different paths. The black lines connected two subpopulations, which contained shared TCRs. Source data are provided as a Source Data file.

To delineate the hierarchy and development relationship between subsets, pseudotime analysis^31^ was performed. The findings unexpectedly revealed two effector differentiation pathways (termed Path I and II) in both PB and BM (Fig. 3a). Signature genes for Pre-branch (such as *TCF7*, *EEF1B2*, *C1orf162*, and *SNHG7*), Path I (such as *PTPRC*, *DDX17*, *MALAT1*, and *PDE3B*), and Path II (such as *HLA-DR*, *LGALS1/3* and *CD74*) were identified (Fig. 3b, Supplementary Data 8). Correlation analysis suggested a conservation between PB and BM of Pre-branch and Path II, but not of Path I (Supplementary Fig. 5a). Different clusters merged into a process in pseudotime that started with naïve subsets (mostly in the Pre-branch), followed by activated/effector subsets, and ending with the *FOXP3*^hi^ subset (Path I) or *MKI67*^hi^ subset (Path II) (Fig. 3a, bottom, Supplementary Fig. 5b). Even cells within the same subsets could be distributed into the two paths, indicating that this bifurcated differentiation is a dominant rule (Fig. 3a, top). There were some overlapping marker genes between the *FOXP3*^hi^ subset and Path I, or between the *MKI67*^hi^ subset and Path II, consistent with the *FOXP3*^hi^ subset as the terminus of Path I and the *MKI67*^hi^ subsets as the terminus of Path II (Supplementary Fig. 5c). Gene Ontology analysis showed that, in both PB and BM, Pre-branch-enriched genes were associated with translation and protein targeting to the endoplasmic reticulum (ER), Path I-enriched genes were related to cell-cell adhesion and cell aggregation, and Path II-enriched genes played roles in response to immune system processes (Fig. 3c, Supplementary Fig. 5d, e, Supplementary Data 9). GSVA indicated that Path II cells expressed higher TCR, activation, suppression, migration, apoptosis, S and G2/M gene sets than did Path I cells (Fig. 3d). Path I cells preferentially expressed FAO gene sets, and Path II cells predominantly expressed glycolysis and TCA gene sets (Fig. 3d).

Inspecting of individual genes revealed that the Path I terminus highly expressed the critical membrane-associated suppressor genes *IL2RA* and *CTLA4* and soluble suppressor genes *FGL2* and *PRF1*. The Path II terminus highly expressed the soluble suppressor genes *LGALS1*, *TGFB1*, *GZMA*, *IL10*, and *IL12A* and the proliferation genes *TOP2A*, *PCNA*, and *HMGB2*, with a slightly higher proportion of S + G2/M cells (Fig. 3e, Supplementary Fig. 6a).

TCR analyses revealed that most of the amplified TCR clonotypes were in Path II cells (Fig. 3f, Supplementary Data 7), corroborating the high glycolysis-proliferation gene signature. Shared TCRs were mostly observed between Pre-branch and Path II and between Path II and Path I, indicating a dominant differentiation from Pre-branch to Path II and the transition between Path II and Path I cells (Fig. 3g). A more detailed analysis of TCR overlap indicated that most Path I *HLA-DR*^hi^ and *FOXP3*^hi^ subset of cells might be derived from the Path II *HLA-DR*^hi^ subset. While Path II *MKI67*^hi^ subset did not transit into Path I cells (Fig. 3h), suggesting that the *MKI67*^hi^ subset was probably a distinctively developed subpopulation.

### In vitro assay of two differentiation Paths

**Fig. 4:**
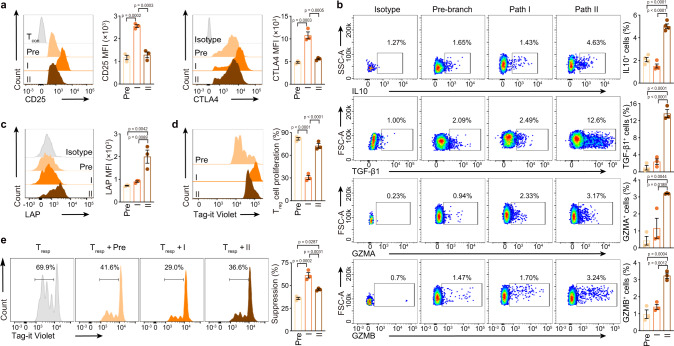
In vitro assay of T_reg_ cell paths. **a**−**c** Flow cytometry and its quantification of the indicated proteins in each HD PB T_reg_ cell path (CD25: *n* = 3, CTLA4: *n* = 3, IL10: *n* = 4, TGF-β1: *n* = 3, GZMA: *n* = 3, GZMB: *n* = 3, LAP: *n* = 3). **d** The proliferation of HD PB T_reg_ cell paths. The Tag-it Violet dilution of T_reg_ cells were assessed by flow cytometry, after 96 h in vitro culture (*n* = 3). **e** In vitro suppression assay (*n* = 3). Tag-it Violet-labeled HD PB T_resp_ cells were co-cultured with different HD PB T_reg_ cell paths for 96 h with CD3/CD28 T cell activator. The T_reg_: T_resp_ ratio was 1: 2. The Tag-it Violet dilution of T_resp_ cells were assessed by flow cytometry. In **a**–**e**, experiment repeated at least three times; p values were determined by One-way ANOVA; data are presented as mean values ± SEM. Source data are provided as a Source Data file.

The surface markers of different paths were identified to validate the properties of the two paths (Supplementary Fig. 6b, c, Supplementary Data 8). In vitro assays were performed after isolation of Path I and II cells by flow cytometry: Pre-branch as CCR7^+^, Path I as CCR7^−^CCR4^med/hi^, Path II as CCR7^−^CXCR3^+^ (Supplementary Figs. 6d, 12a). Path I cells expressed more CD25 and CTLA4 proteins, whereas Path II cells expressed more IL10 and TGF-β1 proteins (Fig. 4a, b, Supplementary Fig. 12a, d). However, the expression of GZMA, GZMB, and LAP (the N-terminal dimer of latent TGF-β1)^32^ proteins were overall very low and only marginally different between Path II and Path I cells (Fig. 4b, c, Supplementary Fig. 12a, d). Path I cells had stronger suppressive but weaker proliferative capacity than Path II and Pre-branch cells in vitro (Fig. 4d, e, Supplementary Fig. 12a–c). Path II cells displayed lower expression of FOXP3 protein than Path I cells, and were slightly larger in size than Pre-branch and Path I cells (Supplementary Figs. 6e, f, 12a, c). Thus, single-cell transcriptomics in combination with functional assays defined two effector differentiation paths in T_reg_ cells.

### Transcription factors enriched in two differentiation paths

**Fig. 5:**
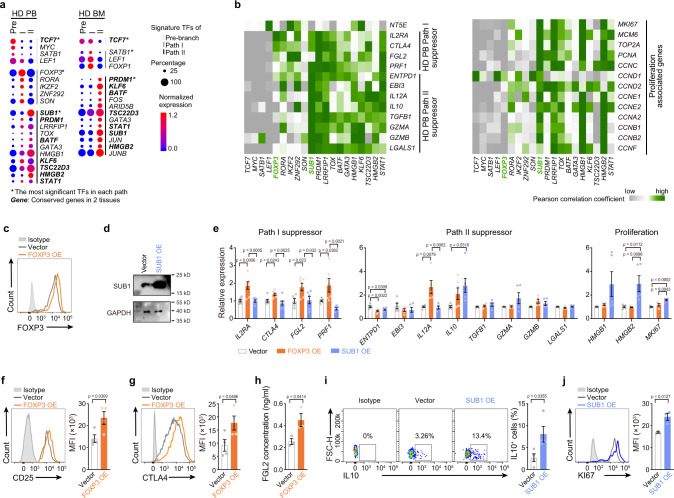
Transcription factors enriched in T_reg_ cell differentiation paths. **a** Dot plots showing the transcriptional factors (TFs) which were differently expressed in each T_reg_ cell path (p < 0.05, log FC > 0.28), ordered by p value. The dot size represents the percentage of cells that express the indicated genes, and the dot color shows the averaged level of expression. FC: fold change. **b** Correlograms visualizing the correlation of single-cell gene expression profiles between TFs and suppression or proliferation genes in HD PB samples. **c** FOXP3 protein levels in HD PB T_reg_ cells, 5 days after transfection with FOXP3 overexpression virus, as measured by flow cytometry. Experiment repeated at least three times. **d** SUB1 protein levels in HD PB T_reg_ cells, 5 days after transfection with SUB1 overexpression virus, as measured by western blot. Experiment repeated three times. **e** Expression of the mRNA level of indicated genes in HD PB T_reg_ cells, 5 days after transfection with FOXP3 or SUB1 overexpression virus, as measured by RT-qPCR (*n* = 5–7). Experiment repeated at least three times. **f**–**j** Expression of CD25, CTLA4, FGL2, IL10 and KI67 in HD PB T_reg_ cells, 5-6 days after transfection with FOXP3 or SUB1 overexpression virus, as measured by flow cytometry (**f**, **g**, **i**, **j**) or ELISA (**h**) (CD25: *n* = 4, CTLA4: *n* = 4, FGL2: *n* = 3, IL10: *n* = 4, KI67: *n* = 3). Experiment repeated three times. In **e**, p values were determined by One-way ANOVA; In **f**–**j**, p values were determined by two-sided unpaired *t*-test; data are presen*t*ed as mean values ± SEM. Source data are provided as a Source Data file.

To understand the transcription factor basis of bifurcated differentiation, the signature transcription factors were identified for each path in PB T_reg_ cells. *TCF7*, *FOXP3*, and *SUB1* were the most significant signature transcription factors in Pre-branch, Path I, and Path II cells from PB, respectively (by p value, Fig. 5a, Supplementary Fig. 6g). PB-BM conservations were evident in the enrichment of *TCF7* in the Pre-branch and *SUB1* and *HMGB2* in Path II (Fig. 5a). Single-cell correlation analysis indicated that *FOXP3* was preferentially co-expressed with Path I-associated suppressor genes *IL2RA*, *CTLA4*, and *FGL2* within individual cells, whereas *SUB1* was frequently co-expressed with Path II-associated suppressor genes *EBI3*, *IL12A*, *IL10*, *TGFB1*, *GZMA*, *GZMB*, and *LGALS1* and with the proliferative genes *MKI67*, *MCM6*, *TOP2A*, *PCNA* and *CYCLIN*s in healthy donor PB (Fig. 5b). A similar gene correlation pattern was observed in the BM (Supplementary Fig. 6h).

To determine whether FOXP3 and SUB1 contribute to the Path I and II features, gain-of-function experiments were performed using T_reg_ cells that overexpressed FOXP3 or SUB1. FOXP3 and SUB1 were successfully overexpressed in T_con_ and T_reg_ cells by infection with recombinant lentivirus (Fig. 5c, d, Supplementary Figs. 7a–d, 12d, e). FOXP3 overexpression increased *IL2RA*, *CTLA4*, *FGL2*, *PRF1*, and *IL12A* mRNA levels. SUB1 overexpression increased *IL10*, *HMGB2*, and *MKI67* mRNA levels in T_reg_ cells (Fig. 5e, Supplementary Fig. 12e). The expression of some genes was confirmed at the protein level. FOXP3 overexpression slightly increased the level of CD25, CTLA4, and FGL2 proteins in T_reg_ cells. SUB1 overexpression increased the level of IL10 protein and slightly increased KI67 protein T_reg_ cells (Fig. 5f–j, Supplementary Fig. 12d, e). The levels of PRF1 and IL12 p35 were overall low in T_reg_ cells and not significantly affected by FOXP3 overexpression (Supplementary Figs. 7e, f, 12d, e). FOXP3 overexpression also significantly promoted the RNA and protein levels of CD25 and CTLA4 in T_con_ cells, consistent with a previous report^33^ (Supplementary Figs. 7g, h, 12d, e). Some other genes that changed after FOXP3/SUB1 overexpression in T_con_ cells were not significantly altered in T_reg_ cells (Supplementary Figs. 7g, 12e), suggesting that these transcription factors may have cell type-dependent functions. These results collectively indicated the possible contribution of FOXP3 and SUB1 to the features of the two paths. However, the changes in some genes were slight after FOXP3 and SUB1 overexpression, suggesting that other transcription factors may also be involved, which warrant further study.

### T_reg_ subsets in allo-HSCT patients with or without aGVHD

**Fig. 6:**
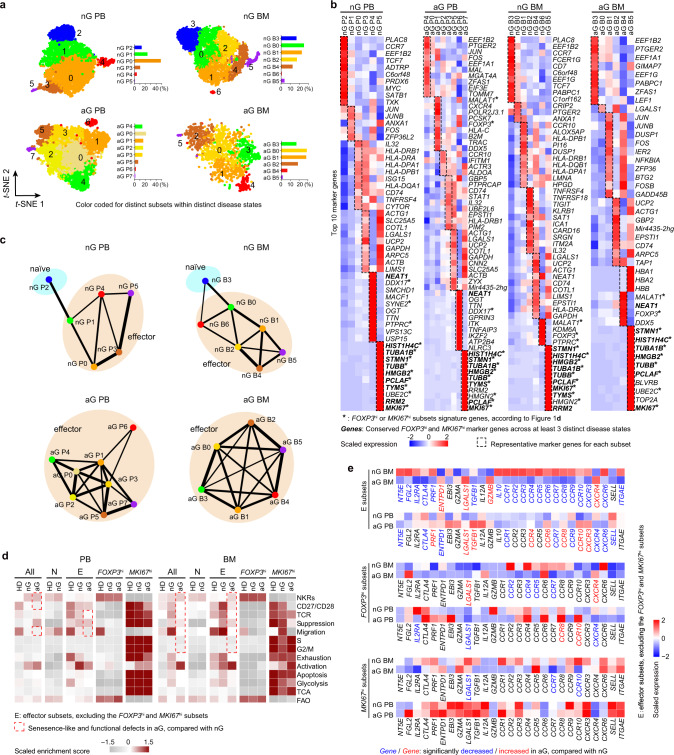
T_reg_ subsets in allo-HSCT patients with or without aGVHD. **a** *t*-SNE of single-cell transcriptomes of T_reg_ cells from allo-HSCT patients, numbers denote subsets. The insert histograms denote the percentage of each subset within specific patients. nG PB/BM, PB/BM samples from non-aGVHD patients; aG PB/BM, PB/BM samples from aGVHD patients (nG PB: *n* = 5, nG BM: *n* = 4, aG PB: *n* = 6, nG BM: *n* = 3). **b** Heatmaps showing the top10 (by fold change) marker genes for each subset, excluding the ribosomal and mitochondrial genes. **c** Partition graph abstraction (PAGA) analysis. Nodes represent clusters, and thicker edges indicate stronger connectedness between clusters. **d** Heatmaps showing the GSVA enrichment score of selected pathways for each subset. **e** Heatmaps showing the expression of migration and suppression-associated genes in different T_reg_ cell subsets. The blue font indicated that the genes were significantly down-regulated in aGVHD patients, compared with non-aGVHD patients (*p* < 0.05). The red font indicated that the genes were significantly up-regulated in aGVHD patients, compared with non-aGVHD patients (*p* < 0.05). In **e**, gene normalized expressions were used and *p*-values were determined by Wilcoxon rank-sum test. Source data are provided as a Source Data file.

T_reg_ cells are largely regenerated after allo-HSCT, but their function and number are impaired if complicated with aGVHD (Supplementary Fig. 8a)^15,34–36^. To understand the disturbance of T_reg_ subsets and pathways under this pathological condition, scRNA-seq was used to explore *FOXP3*^+^ T cells from the PB and BM of allo-HSCT patients with or without aGVHD (Supplementary Data 1). Chimerism analyses showed early reconstitution of donor T cells (> 96%, Supplementary Data 1). T_reg_ subsets were resolved and compared with healthy donor T_reg_ atlas, which revealed that naïve subsets (non-aGVHD P2, non-aGVHD B3), *FOXP3*^hi^ subsets (non-aGVHD P4, aGVHD P6, non-aGVHD B6, aGVHD B4) and *MKI67*^hi^ subsets (non-aGVHD P5, aGVHD P7, non-aGVHD B5, aGVHD B5) were present in allo-HSCT patients regardless of primary diseases (Fig. 6a–c, Supplementary Fig. 8b–d, Supplementary Data 4). Other effector clusters in allo-HSCT patients were not highly correlated with any effector clusters in healthy donors, indicating that dramatic changes occurred in effector subpopulations after allo-HSCT. Compared with healthy donors, allo-HSCT patients without aGVHD displayed reduced naïve T_reg_ cells, whereas aGVHD patients almost completely lost naïve T_reg_ cells (Fig. 6a–c, Supplementary Fig. 8e). In the BM, the *MKI67*^hi^ subset was expanded up to 3% in non-aGVHD patients (non-aGVHD B5), but was decreased to < 1% (aGVHD B5) in aGVHD patients (Fig. 6a). These results indicated that *FOXP3*^hi^ and *MKI67*^hi^ subsets still preserved their identity, but other effector T_reg_ cells had undergone dramatic alterations after allo-HSCT.

GSVA analysis of T_reg_ subsets indicated that the effector T_reg_ subsets from non-aGVHD patients had slightly higher expression of suppression-associated genes than those from healthy donors (Fig. 6d). Even though *FOXP3*^hi^ or *MKI67*^hi^ subsets seemed to be comparable between non-aGVHD and aGVHD conditions, other effector T_reg_ cells from PB and BM of aGVHD patients displayed lower expression of suppression-, migration-, and TCR-associated gene sets, compared with the non-aGVHD patients (Fig. 6d). In the BM of aGVHD patients, the effector T_reg_ subsets (excluding the *FOXP3*^hi^ and *MKI67*^hi^ subsets) displayed a senescence-like defect characterized by high expression of natural killer cell receptors (NKRs) and lower expression of CD27/CD28^37–39^ (Fig. 6d). These defects were not associated with an exhausted phenotype (Fig. 6d). As the aGVHD patients in this study were relatively older than non-aGVHD patients (Supplementary Fig. 8f), the senescence-like defect might be attributed to the age of aGVHD patients.

Examination of individual genes related to suppression and migration revealed that effector T_reg_ cells (excluding the *FOXP3*^hi^ and *MKI67*^hi^ subsets) from non-aGVHD patients had higher levels of *IL2RA*, *ENTPD1*, *EBI3*, *LGALS1*, and *CCR1/3/5* than with those from healthy donors (Supplementary Fig. 9). *FOXP3*^hi^ or *MKI67*^hi^ subsets displayed few genes that were differentially expressed between non-aGVHD and aGVHD conditions (Fig. 6e). Effector T_reg_ cells from aGVHD patients had lower levels of *NT5E*, *CTLA4*, *CCR1*, *CCR7*, *CCR9*, and *CXCR6* than those from non-aGVHD patients (Fig. 6e). In addition, lower levels of other genes such as *FGL2*, *IL2RA*, *PRF1*, *TGFB1*, *IL10*, *CCR2-6*/*8*/*10*, *CXCR3*, and *ITGAE* in BM effector T_reg_ cells were found in aGVHD patients than in non-aGVHD patients (Fig. 6e), indicating severe defects in BM T_reg_ cells. These results suggested that the *FOXP3*^hi^ and *MKI67*^hi^ subsets were stable populations in allo-HSCT patients regardless of aGVHD, whereas the other effector subsets underwent dramatic changes in frequency, signature, and function under allo-HSCT and aGVHD conditions.

### T_reg_ paths in allo-HSCT patients with or without aGVHD

**Fig. 7:**
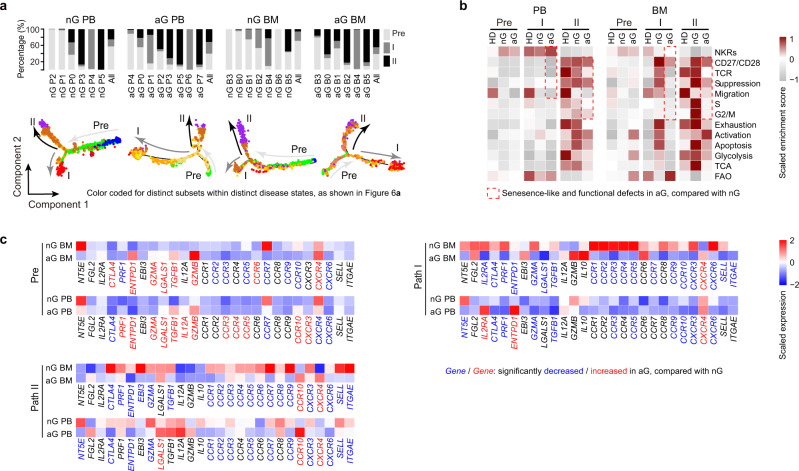
T_reg_ Paths in allo-HSCT patients with or without aGVHD. **a** Trajectory analysis for T_reg_ cell clusters in allo-HSCT patients, colored by subsets (nG PB: *n* = 3, nG BM: *n* = 3, aG PB: *n* = 4, nG BM: *n* = 3). The insert picture shows the proportions of different T_reg_ cell paths in each subset. **b** Heatmaps showing the GSVA enrichment score of selected pathways for each T_reg_ cell path. **c** Heatmaps showing the expression of migration and suppression-associated genes in T_reg_ Paths. The blue font indicated that the genes were significantly down-regulated in aGVHD patients, compared with non-aGVHD patients (*p* < 0.05). The red font indicated that the genes were significantly up-regulated in aGVHD patients, compared with non-aGVHD patients (*p* < 0.05). In **c**, gene normalized expressions were used and p values were determined by Wilcoxon rank-sum test. Source data are provided as a Source Data file.

T_reg_ cells from allo-HSCT patients preserved the two differentiation paths, mostly with the *FOXP3*^hi^ subset at the Path I terminus (except non-aGVHD BM), and with the *MKI67*^hi^ subset at the Path II terminus (Fig. 7a, Supplementary Fig. 10a, Supplementary Data 8). Allo-HSCT patients also expressed high levels of suppressor genes *LGALS1* and cell cycle genes *TOP2A*, *PCNA*, and *MCM6* in Path II, and expressed high levels of suppressor genes *IL2RA*, *CLTA4*, and *FGL2* in Path I, similar to healthy donor T_reg_ cells (Supplementary Fig. 10b). GSVA demonstrated that in allo-HSCT patients, Path II still had higher TCR signaling, suppression, migration, cell cycle, apoptosis, glycolysis, and TCA than Path I, similar to the observations in healthy donors (Fig. 7b). These results suggested that the two-path differentiation modes were preserved in allo-HSCT patients regardless of aGVHD.

There were also alterations in the paths after allo-HSCT. Compared with healthy donors, non-aGVHD patients had higher levels of *IL2RA*, *ENTPD1*, *GZMA*, *LGALS1*, *CCR3*, *CCR5*, and *CXCR6*, and lower levels of *CCR7*, in Pre-branch, Path I, and Path II. These findings might suggest a general T_reg_ functional activation after allo-HSCT (Supplementary Fig. 10c). GSVA analysis revealed that, compared with non-aGVHD patients, aGVHD patients displayed senescence-like defects and reduced suppression and migration capacity (Fig. 7b) in both Path I and Path II. Examination of the individual genes revealed that, compared with non-aGVHD patients, aGVHD patients expressed lower levels of *CCR7* and *CXCR6* in Pre-branch, lower levels of *CTLA4*, *PRF1*, *GZMA*, *TGFB1*, *CCR3*, *CCR5*, *CCR9*, *CCR10*, *CXCR3*, and *CXCR6* in Path I, and lower levels of *CTLA4*, *ENTPD1*, *GZMA*, *CCR1/2/3/5/7/9*, *CXCR3*, *CXCR6*, *SELL*, and *ITGAE* in Path II (Fig. 7c). These results suggested that aGVHD T_reg_ cells have displayed path-specific defects in suppression and migration.

## Discussion

Multi-faceted investigations of T_reg_ heterogeneity have revealed diverse classifications, which largely improved the knowledge of T_reg_ cells and potential clinical benefits. The present single-cell transcriptome-based exploration of human T_reg_ cells from healthy donors and stem cell transplanted patients corroborates previous findings and brings some fresh insights into T_reg_ cell biology. We resolved healthy donor PB and BM T_reg_ cells into nine subsets continuously spanning naïve and activated/effector stages. Among them, *FOXP3*^hi^ and *MKI67*^hi^ subsets were highlighted. The *FOXP3*^hi^ subset had the highest expression of *FOXP3* and *IL2RA*. The *MKI67*^hi^ subset was characterized by high proliferation-associated genes. *FOXP3*^hi^ and *MKI67*^hi^ subsets had the strongest suppressive capacity, suggesting that they might be important in vivo despite their limited numbers. Although the *FOXP3*^hi^ subset did not exhibit a remarkably higher score than other effector subsets in GSVA analysis based on all suppressor genes, it expressed the highest levels of *IL2RA* and *CTLA4* (in PB), which are two critical mediators of T_reg_ suppressor function^3^ and might account for its superior suppressive capacity. Compared with the highly conserved *FOXP3*^hi^ and *MKI67*^hi^ subsets, the overall signature of the *HLA-DR*^hi^ or *LIMS1*^hi^ subsets were less conserved between PB and BM. The *HLA-DR*^hi^ subset displayed the highest degree of activation, which has been described previously^40^. The *LIMS1*^hi^ subset lost FOXP3 expression when cultured in vitro, but this result could be more or less affected by the presence of FOXP3-negitive cells in this sorted T_reg_ cell subset (Supplementary Fig. 4a). Unexpectedly, in our study, *LIMS1*^hi^ cells still retained strong suppression ability (Fig. 2e), probably due to FOXP3-independent mechanisms or because the residual FOXP3^+^ cells can still exert a strong suppressive effect. The *LIMS1*^hi^ subset somehow resembled the inducible murine T_reg_ cells mentioned in a previous study^41^. The findings of a recent study that used scRNA-seq to map umbilical cord blood T_reg_ cells in an inflammatory setting and suggested that the TIGIT^−^ subset was sensitive to IL6 and developed an “unstable” T_reg_ identity^42^. This subset is similar with the *LIMS1*^hi^ subset in terms of low expression of *TIGIT* (Supplementary Fig. 11a). However, there was no evidence in the current study concerning whether the human *LIMS1*^hi^ cells were induced or unstable T_reg_ cells. The relationship between our *LIMS1*^hi^ subset and the previously described TIGIT^−^ subset in literature awaits future study. In a previous study using mass cytometry, 22 T_reg_ subpopulations were identified in the PB of human^7^. The CD45RA^+^CCR4^−^CD31^+^, CXCR3^+^CD38^low^ICOS^low^, and CXCR3^−^CD38^+^ subpopulations in their study were similar to our naïve subsets (P0, 3, 5), P6 effector subset, and P4 effector subset, respectively (Supplementary Fig. 11b). However, as mRNA levels may not always be consistent with protein levels, a very strict comparison is difficult. Future analysis of protein and mRNA in the same cells may clarify the relationship between the identified subpopulations in different studies.

Our results also reveal two disparate differentiation pathways in T_reg_ cells. Path I had high expression of CD25 and CTLA4, and genes associated with FAO and was termed as the *FOXP3*^hi^ subset. In contrast, Path II had high expression of IL10 and TGF-β1, and genes associated with glycolysis and proliferation and was termed as the *MKI67*^hi^ subset. Intriguingly, even cells within the same effector cluster could be divided into two paths, suggesting that the bifurcated differentiation is a dominant rule that governs T_reg_ cells. Single-cell TCR analysis can provide in-depth information about T_reg_ cell differentiation. A previous single-cell analysis indicated the close relationship of mouse T_reg_ cells with shared TCRs^12^. Presently, high-frequency TCR clonotypes were mainly detected in activated/effector T_reg_ cells, suggesting that the acquisition of the activation/effector phenotype requires clonal expansion. Effector T_reg_ subsets had the highest numbers of overlapping TCR clonotypes, suggesting their frequent transition. Surprisingly, the *FOXP3*^hi^ and *MKI67*^hi^ subsets had hardly any overlapping clonotypes, and the *MKI67*^hi^ subset did not contain repeated TCRs. This may reflect their distinct developmental mode or be attributed to the few cells that were collected. Overlapping TCR clonotypes were abundant between Pre-branch and Path II and between Path II and Path I but were rare between Pre-branch and Path I, suggesting a probable differentiation route from Pre-branch to Path II and the transition between Path II and Path I cells. However, we should not rule out the possibility of differentiation from Pre-branch to Path I. We also explored the transcription factors that may contribute to Path I or Path II phenotype. Notably, the Path I cells displayed high expression of *FOXP3*, whereas Path II cells displayed high expression of *SUB1*. Overexpression of FOXP3 and SUB1 slightly but repeatedly promoted the expression of some Path I- and Path II-associated suppressive genes, respectively, suggesting they may be involved in promoting Path I and Path II phenotypes. However, after FOXP3 and SUB1 overexpression, the gene expression changes were mostly slight, suggesting that the Path I and Path II phenotypes may also be regulated by many other transcription factors that are enriched in Path I and Path II cells. This needs to be further investigated.

T_reg_ cell differentiation has been extensively explored. A recent report indicated that human T_reg_ cells may simulate Th cell differentiation mechanisms and can be divided into Th1, Th17, Th1/17, Th2, and Th22-like subpopulations^8^. Our data suggested that the *FOXP3*^hi^ subset expressed some genes associated with T_reg_2-like cells, whereas the *MKI67*^hi^ subset expressed some genes associated with T_reg_1-like cells (Supplementary Fig. 11c). However, other subsets had more complex features and did not fit well with obvious Th-like gene expression patterns. In addition, the two paths of differentiation revealed by our trajectory analysis did not show a tendency to express a specific Th-like signature (Supplementary Fig. 11d). For example, *TBX21* and *GATA3* were expressed at higher levels in Path I, while *IFNG* was higher in Path II. Additional studies are needed to explore this important issue. A previous study has suggested that T_reg_ cells from non-lymphoid organs differ from those in lymphoid organs and exhibited tissue-adapted gene signatures^13^. The present findings identified differences in gene patterns between PB and BM T_reg_ cells. Future studies on other tissues will further resolve the tissue-specific differentiation of T_reg_ cells.

T_reg_ cell heterogeneity and differentiation were also studied in the context of allo-HSCT. *FOXP3*^hi^ and *MKI67*^hi^ T_reg_ subsets and the two differentiation pathways were still present in allo-HSCT patients regardless of aGVHD. However, other effector subsets from aGVHD patients displayed a senescence-like phenotype, and a decreased expression of suppression and migration-related molecules. Our results are consistent with previous microarray analysis data suggesting reduced expression of migratory and suppressive genes, including *CCR1*, *CCR3*, *CCR5*, *CXCR3*, *CXCR6*, *LGALS1*, and *GZMA*, in aGVHD patients^43^. With the caveat of the examination of a limited number of specimens, the average age of aGVHD patients was higher than that of non-aGVHD patients, although the average age of donors was comparable. A previous study described that the combination of donor and recipient age is a predictor of GVHD^44^. The senescence-like defect of T_reg_ cells from aGVHD patients might be at least partially attributed to the older age of aGVHD patients compared with non-aGVHD patients.

In conclusion, with an unbiased single-cell approach, our study reveals previously unrecognized effector subsets and two-path differentiation structure in the human T_reg_ compartment. These aspects are conserved between PB and BM and between steady state conditions and immune disturbance. The features related to function, migration, metabolism, activation, proliferation, TCR signaling, and transcription factors in different subsets and pathways are illustrated. We also suggest the transcription factor FOXP3 and SUB1 might be involved in promoting some features of the two-paths, although future study on the complete list of differentially expressed transcription factors will provide more complete understanding of two-path differentiation. These findings enrich the knowledge of human T_reg_ cell heterogeneity, function, and differentiation, and provide a single-cell resolution atlas that will inform the greater understanding of T_reg_ cells and T_reg_ cell-related diseases and therapeutic interventions in these diseases.

## Methods

### Human specimens

This study was approved by the Ethics Committee of the State Key Laboratory of Experimental Hematology, Institute of Hematology and Hospital of Blood Disease, Chinese Academy of Medical Sciences & Peking Union Medical College, Tianjin, China (approval number: KT2017069-EC-2). All the people in this study provided written informed consent for sample collection and data analyses. The inclusion criteria of patients were allogeneic stem cell transplantation, donor cell chimerism of CD3^+^ cells > 95%, and no active infections (i.e., cytomegalovirus or hepatitis B virus). Nine HDs, six non-aGVHD patients and six aGVHD patients were enrolled in this study. aGVHD was staged according to modified Glucksberg criteria^45^. Their ages ranged from 12 to 58, with a median age of 32. All HSCT patients received aGVHD prophylaxis with tacrolimus or ciclosporin plus short-term methylprednisolone, with or without ruxolitinib. PB samples with/without paired BM samples were obtained for the subsequent lymphocyte isolation. Patient characteristics are given in Supplementary Data 1.

### Specimen preparation of single-cell suspensions

PB and BM were collected, and coagulation was prevented by the addition of 50 U/ml heparin (Sigma-Aldrich, MO, USA). Mononuclear cells from PB or BM were isolated using Lymphoprep (STEMCELL, Vancouver, Canada) according to the manufacturer’s instructions. Lysis of red blood cells was performed with 500 μl of ACK (Lonza, NJ, USA) for 5 min on ice. These cells were resuspended in sorting buffer PBS supplemented with 1% fetal bovine serum (FBS, Gibco, CA, USA). Suspensions were passed through a 70 μm cell strainer before immunostaining.

### Flow cytometry

For the analysis of cell surface molecules, single-cell suspensions were prepared and incubated with the following antibodies: PE-Cyanine7-anti-human CD4 (357410, Biolegend, 1/200), BV785-anti-human CD4 (300553, Biolegend, 1/200), APC-Cyanine7-anti-human CD3 (317341, Biolegend, 1/200), APC-anti-human CD25 (302610, Biolegend, 1/100), BV711-anti-human CD25 (356137, Biolegend, 1/100), FITC-anti-human CD127 (351312, Biolegend, 1/100), BV510-anti-human CD127 (351331, Biolegend, 1/100), BV650-anti-human CCR7 (353234, Biolegend, 1/100), APC-anti-human CCR7 (353213, Biolegend, 1/100), BV605-anti-human HLA-DR (307639, Biolegend, 1/100), APC-anti-human TIGIT (372706, Biolegend, 1/50), APC-Cyanine7-anti-human ITGA4 (304328, Biolegend, 1/50), PerCP-Cyanine5.5-anti-human CCR4 (359405, Biolegend, 1/100), APC-anti-human CCR4 (359407, Biolegend, 1/100), PE-Cyanine7-anti-human CD38 (356608, Biolegend, 1/100), FITC-anti-human CD59 (304706, Biolegend, 1/100), PE-anti-human CD59 (304707, eBioscience, 1/100), PE-anti-human CXCR3 (2009783, Invitrogen, 1/100), FITC-anti-human CXCR3 (353704, Biolegend, 1/50), PE/Cy7-anti-human CD161 (339917, Biolegend, 1/100), PE-anti-human CD152 (369603, Biolegend, 1/100), PE-Cyanine7-anti-human CD152 (369613, Biolegend, 1/100), APC-mouse IgG1 (400119, Biolegend, 1/200), APC-Cyanine7-mouse IgG1 (400127, Biolegend, 1/200), PE-mouse IgG1 (400111, Biolegend, 1/200), PE/Cy7-mouse IgG1 (400125, Biolegend, 1/200), PerCP/Cyanine5.5-mouse IgG1 (400149, Biolegend, 1/200), FITC-mouse IgG1 (400107, Biolegend, 1/200), and BV421-mouse IgG1 (400157, Biolegend, 1/200). Intracellular staining of FOXP3 (PE, 320107, Biolegend, 1/100 or PE, 4331087, eBioscience, 1/50), cytokines and other proteins (FITC-anti-human Helios, 137204, Biolegend, 1/100; APC-anti-human LAP, 349705, Biolegend, 1/100; BV421-anti-human KI67, 562899, BD Bioscience, 1/100) was performed with FOXP3 staining kits (eBioscience, CA, USA). To detect the expression of cytokines IL10 (PerCP/Cyanine5.5, 501417, Biolegend, 1/50), TGF-β1 (BV421, 562962, BD Bioscience, 1/50), GZMA (PE-Cyanine7, 25-9177-41, eBioscience, 1/100), GZMB (FITC, 515403, Biolegend, 1/100), Perforin (BV510, 308119, Biolegend, 1/50), and IL12 p35 (eFluor® 660, 50-7359-41, Invitrogen, 1/50) cells were stimulated with phorbol myristate acetate (PMA, 50 ng/ml) and ionomycin (500 ng/ml) in the presence of GolgiStop (BD Biosciences) for 4 h before analysis^46^. The antibodies were obtained from eBioscience, Biolegend (CA, USA), Invitrogen and BD Biosciences (CA, USA) and were listed in Supplementary Data 10. Flow cytometry data were acquired on LSR II, FACSCanto II or FACSAria III (BD Biosciences) and analyzed with FlowJo software (Tree Star, OR, USA).

### Single-cell sorting and processing of 10× Genomics single cell RNA-seq and TCR-seq

Based on FACS analysis, single cells of different subtypes, including DAPI^−^CD3^+^CD4^+^CD25^+^CD127^−^ T_reg_ cells and DAPI^−^CD3^+^CD4^+^CD25^−^ T_con_ cells, were sorted into microcentrifuge tubes (Axygen, CA, USA) filled with 200 µl of PBS with 0.06% BSA. Cells were then encapsulated in one lane of a 10× Chromium instrument, and libraries were constructed with a Chromium Single Cell 3ʹ GEM, Library & Gel Bead Kit v3 or Chromium Single Cell 5′ Library & Gel Bead Kit plus Chromium Single Cell V(D)J Enrichment Kit (Human T Cell), following the 10× Genomics protocol (10× Genomics, CA, USA).

### Processing and quality control of single-cell RNA-seq data

For 3′ single cell RNA-seq and 5′ single cell RNA-seq data, raw reads obtained from the 10× Genomics single-cell RNA-seq platform were demultiplexed and mapped to the human reference genome GRCh38 using the CellRanger software (version 3.0.2) (https://support.10xgenomics.com/single-cell-gene-expression/software) with default parameters. Cells were removed if they expressed fewer than 200 unique genes, or greater than 20% mitochondrial genes. Then, genes that were expressed in 5 or more cells were retained for further analysis. FOXP3 is essential for T_reg_ cells to function properly. In this study, *FOXP3* positive cells were retained for downstream analysis. Finally, our study included 43,178 *FOXP3*^+^ T_reg_ cells and 3,138 *CD4*^+^*FOXP3*^−^ T_con_ cells (Supplementary Data 2).

### Dimensionality reduction and cell clustering

The R package Seurat (version 3.0.2)^22^ implemented in R (version 3.6) were used to perform dimensional reduction of T_reg_ and T_con_ cell RNA data. The “*NormalizeData*” function from Seurat was used to normalize the raw counts, and the scale factor was set to 100,000, then followed by “*FindVariableFeatures*” with default parameters to calculate highly variable genes for each sample. After performing “JackStraw”, which returned the statistical significance of PCA scores, we selected ten significant PCs to conduct dimension reduction and cell clustering. Then, cells were projected in 2D space using *t*-SNE or UMAP with default parameters. Clustering on individual tissues used the following resolutions: for T_reg_ cells, 0.5 on HD-BM, 0.5 on HD-PB, 0.4 on non-aGVHD-BM, 0.36 on non-aGVHD-PB, 0.36 on aGVHD-BM, 0.5 on aGVHD-PB; for T_con_ cells 0.3 on HD-PB.

### Integrated analysis of different conditions 10× Genomics-derived data

To account for batch effect among different samples in each condition (HD-BM, HD-PB, Non-aGVHD-BM, Non-aGVHD-PB, aGVHD-BM, aGVHD-PB), we used “*FindIntegrationAnchors*” in the Seurat package to remove batch effect and merge samples in each condition to one object. In detail, the top 2 000 genes with the highest expression and dispersion from each sample were used to find the integration anchors, and then the computed anchoret was applied to perform dataset integration.

### Identification and analysis of differentially expressed genes

To identify unique differentially expressed genes (DEGs) among each cluster, the “*FindAllMarkers*” function from Seurat was used and non-parametric Wilcoxon rank sum tests were set to evaluate the significance of each individual DEG. The DEGs with adjusted *P* value less than 0.05 were thought to be significant and used in downstream analysis. Then, significant genes were selected as input to perform gene ontology analysis through DAVID (https://david-d.ncifcrf.gov, version 6.8)^47^.

### Clustering, heatmaps and dot plots for gene expression in single cells

Hierarchical clustering and heatmap generation were performed for single cells on the basis of normalized expression values of marker genes curated from the literature or identified significant DEGs. To visualize the expression of individual genes, cells were grouped by their cell type as determined by analysis with Seurat. Normalized gene expression values were plotted for each cell type as a heatmap or dot plot in R.

### PAGA analysis

To assess the global connectivity topology between the T_reg_ cell clusters we applied Partition-based graph abstraction (PAGA)^48^. The weighted edges represent a statistical measure of connectivity between the partitions. Connections with a weight less than 0.3 were removed.

### Functional enrichment analysis of signature genes

Gene set variation analysis (GSVA) analysis was performed to identify the pathway alterations that underlie our T_reg_ cell subsets with the Bioconductor package GSVA (version 1.16.0). The expression matrix of T_reg_ cells were subjected to the GSVA algorithm to calculate GSVA enrichment scores for each gene set, and the gene sets are listed in Supplementary Data 6.

### Pseudotime trajectories analysis

Pseudotime trajectories were constructed with the R package Monocle (version 2.12.0)^49^, ordering genes were identified by the “*differentialGeneTest*” function, and adjusted *P* value less than 0.001 were regarded as significant and used to order cells. The discriminative dimensionality reduction with trees (DDRTree) method was used to reduce data to two dimensions and visualized through the “*plot_cell_trajectory*” function. To detect genes that play essential roles in cell fate decisions, branched expression analysis modeling (BEAM) from Monocle was implemented to identify genes with branch-dependent expression and visualized with the “*plot_genes_branched_heatmap*” function.

### Preprocessing and analysis of scTCR-seq data

TCR sequence data from Chromium single cell 5′ RNA-seq libraries were processed by CellRanger (version 3.0.2) with default parameters. To compare the TCR data among different samples, multiple libraries were analyzed together according to the documentation provided by 10× Genomics (https://support.10xgenomics.com/single-cell-vdj/software/pipelines/latest/advanced/multi-library-samples). First, we constructed a mro file and checked by “*cellranger mrc*”. Then, “*cellranger vdj*” was used to align TCR data to human Cell Ranger V(D)J compatible reference (http://cf.10xgenomics.com/supp/cell-vdj/refdata-cellranger-vdj-GRCh38-alts-ensembl-3.1.0). The number of distinct UMIs aligned to each TCR alpha/beta pair less than 10 were filtered out, and only the productive TCR alpha/beta pairs were kept for further analysis. Finally, we identified the TCR alpha/beta pairs for 13,349 cells.

### Isolation of T_reg_ clusters or different path cells

T_reg_ cell clusters or paths were purified from human PBMCs with the indicated genotypes. In brief, CD4^+^ T cells or CD4^+^CD25^+^CD127^low^ T_reg_ cells were selected from cell suspensions by EasySep™ Human CD4^+^ T Cell Isolation Kit or EasySep™ Human CD4^+^CD127^low^CD25^+^ Regulatory T Cell Isolation Kit (STEMCELL) according to the manufacturer’s instructions and then isolated as by FACSAria III (BD Biosciences). Living T_reg_ cluster cells (DAPI negative, CD4^+^CD25^+^CD127^−^) were isolated with the following marker antibody combinations: HLA-DR^−^CCR7^++^TIGIT^−^ for cluster 5 (P5), HLA-DR^−^CCR7^+^TIGIT^−^ for cluster 3 (P3), HLA-DR^−^CCR7^+^TIGIT^+^ for cluster 0 (P0), HLA-DR^−^CCR7^−^TIGIT^+^ITGA4^−^ for cluster 1 (P1), HLA-DR^−^CCR7^−^TIGIT^+^ITGA4^+^ for cluster 6 (P6), HLA-DR^−^CCR7^−^TIGIT^−^ for cluster 4 (P4), HLA-DR^+^CCR7^−^CD25^++^CCR4^++^ for cluster 7 (P7), HLA-DR^+^CCR7^−^CD38^+^CD59^+^ for cluster 8 (P8), and HLA-DR^+^CCR7^−^CD38^−^CD59^−^CD25^+^CCR4^+^ for cluster 2 (P2). Living T_reg_ different Path cells (DAPI negative, CD4^+^CD25^+^CD127^−^) were isolated with marker antibody combinations as follows: as CCR7^+^ for Pre-branch, CCR7^−^CCR4^med/hi^ for Path I, and CCR7^−^CXCR3^+^ for Path II.

### In vitro T_reg_ suppression assay, cell lineage maintenance and survival

Based on FACS analysis, DAPI^−^CD4^+^CD25^−^CD44^−^CD62L^+^ T cells were sorted and used as responder cells (T_resp_). For in vitro suppression assay, T_resp_ cells (4 × 10^4^) were labeled with Tag-it Violet^TM^ proliferation and cell tracking dye kit (Biolegend) and cultured in the presence or absence of FACS-isolated T_reg_ cells from distinct clusters or different path cells with the ratio 1: 2 (T_reg_: T_resp_) for 96 h, with CD3/CD28 T cell activator (3 µl/ml, STEMCELL). The cell division index of T_resp_ cells was assessed by dilution of Tag-it Violet, using FlowJo software. The calculation formula to determine the suppression ability of T_reg_ in vitro is: Suppression (%) = (Percentage of proliferating T_resp_ cells alone - Percentage of proliferating T_resp_ cells treated with T_reg_)/ Percentage of proliferating T_resp_ cells alone × 100^50,51^. The in vitro FOXP3 stability, proliferation capacity and apoptosis of T_reg_ cells were assessed after 96 h in vitro culture (3 µl/ml CD3/CD28 T cell activator, 20 ng/ml IL-2) by the expression of FOXP3, the dilution of Tag-it Violet and the expression of Annexin V, respectively.

### Plasmid preparation and lentivirus packaging

To overexpress the indicated TFs, the canonical CDS at each gene locus was obtained from NCBI GenBank. After necessary sequence optimization for gene cloning, DNA sequences were synthesized and sub-cloned into the lentivirus vector pLVX-IRES-mCherry (Takara, Tokyo, Japan). The TF overexpression vectors were co-transfected into 293 T (CRL-11268, American Type Culture Collection) cells with the 2^nd^-generation lentivirus packaging plasmids psPAX2 and pMD2G, and the virus in the medium supernatant was harvested and concentrated for target cell infection.

### In vitro cell culture and gene transduction of T_reg_ and T_con_ cells

Human PBMC-derived T_reg_ cells were isolated via the MACS strategy (STEMCELL) based on their surface markers: CD4^+^CD25^+^CD127^−^, and FACS was followed to isolate T_reg_ cells in high purity, with sorting markers: DAPI^−^CD4^+^CD25^+^CD127^−^. In some cases, DAPI^−^CD4^+^CD25^−^ T_con_ cells were also isolated via FACS. T_con_ and T_reg_ cells were maintained in the human T cell growth medium (STEMCELL) and supplemented with IL-2 at concentrations of 100 ng/ml and 500 ng/ml, respectively, and anti-human CD3/CD28 T cell activator (25 µl/ml) were added to the medium to initiate T_con_ or T_reg_ cell proliferation. After 48 h of in vitro activation, lentivirus with a typical titer of 5 × 10^7^ TU/ml was added to the cell culture medium to reach the final MOI of 15, and a cell spin-infection protocol was applied for gene transduction. Polybrene was also added to the medium to enhance the gene transduction efficiency. Eight to ten hours post infection, the cell supernatant was replaced with fresh T cell growth medium to facilitate the removal of virus and transduction reagent from cells. Seventy-two hours later, transduction efficiency was determined by measuring the percentage of mCherry^+^ cells with FACS. 5-6 days after transduction, lentivirus transduced cells were purified by FACS sorting of mCherry^+^ cells. The following RT-PCR, flow cytometry staining and Western blot were carried out in the sorted cells. With the limited cell numbers, we transducing the cells with vector and FOXP3-OE virus or with vector and SUB1-OE virus in the detection of protein levels of genes.

### RNA extraction and RT-qPCR

Lentivirus transduced T_reg_ or T_con_ cells were sorted by FACS and Total RNA was extracted from these sorted cells using an RNeasy Mini Kit (Qiagen), and reverse transcribed into cDNA using a High-Capacity cDNA Reverse Transcription Kit (Applied Biosystems, CA, USA). For semiquantitative PCR, the SYBR green-based CT^ΔΔT^ method was carried out, and all reactions were run on an ABI 7900 instrument. The Primers included in the experiments are listed in Supplementary Data 10, and the 18 S rRNA expression level was selected for the internal reference for target expression level analysis.

### Enzyme-linked immunosorbent assay

Lentivirus transduced T_reg_ cells were sorted by FACS and then cells were stimulated with CD3/CD28 T cell activator (25 µl/ml) for 48 h to amplify FGL2 production. Post stimulation, the conditional medium was collected and cell debris was removed by centrifugation. The soluble form of FGL2 secreted by T_reg_ cells was quantified by LEGEND MAX™ Human FGL2 Enzyme-Linked Immunosorbent Assay (ELISA) Kit (Biolegend). The manufacturer’s instructions were followed to measure the secretory FGL2 levels in conditional medium, and the concentrations of FGL2 were converted from 450nm-570nm absorbance, which the microplate reader detected. The standard curve was plotted at the same time to facilitate the calculation of FGL2 concentrations.

### Western blot

Lentivirus transduced T_reg_ cells were sorted by FACS, and then cells were lysed for SDS-PAGE. After electrophoresis, the denatured protein samples were transferred onto nitrocellulose membrane for incubation with primary antibody. Antibodies to SUB1 (HPA001311, Sigma-Aldrich) and GAPDH (D16H11, Cell Signaling Technology) were both applied with a dilution rate of 1: 3000. Post incubation with the primary antibodies overnight at 4 °C, membranes were washed by TBST, then followed by the other incubation with HRP-conjugated secondary antibody for an hour at room temperature^52^. The specific signals from HRP-ECL reaction were visualized by the ChemiDoc imaging system (Bio-Rad).

### Statistical analysis

Experimental results are reported as mean values ± SEM; *n* represents numbers of patients or healthy donors in the experiments, as specified in the Figure Legends. An unpaired two-tailed Student’s *t*-test (for two group comparisons) or a one-way ANOVA was performed using Prism (GraphPad, CA, USA) and the Wilcoxon rank-sum test was performed using R package ggpubr. A *p*-value of 0.05 was considered statistically significant. No specific randomization or blinding protocols were used. The ANOVA results of certain experiments were given in Supplementary Data 11. Source data are provided as a Source Data file.

### Reporting summary

Further information on research design is available in the Nature Research Reporting Summary linked to this article.

## Supplementary information

Supplementary information

Description of Additional Supplementary Files

Supplementary Data 1-11

Reporting Summary

## Data Availability

The scRNA-seq data and scTCR-seq data sets have been deposited in the Gene Expression Omnibus (GEO) at “GSE175604”. Data usage shall be in full compliance with the Regulations on Management of Human Genetic Resources in China. All other relevant data supporting the key findings of this study are available within the article and its Supplementary Information files or from the corresponding author upon reasonable request. The source data underlying Figs. 1h, 2c-e, 3a, 3f, 4a-e, 5d-j, 6a, 7a and Supplementary Figs. 1c-f, 2e, 3a, 3c, 4a-f, 5b, 6a, 6e, 6f, 7b, 7e–h, 8a, 8c, 8e, 8f are provided as a Source Data file with this paper. Source data are provided with this paper. A reporting summary for this Article is available as a Supplementary Information file. Source data are provided with this paper.

## Source data

Source Data
