# Supplementary material for: Single-cell transcriptomic analysis reveals disparate effector differentiation pathways in human Treg compartment
Source: Nat Commun. 2021 Jun 23;12:3913. doi: 10.1038/s41467-021-24213-6 (PMC8222404; doi:10.1038/s41467-021-24213-6)
Supplement: Supplementary file 1 — Supplementary information [file 41467_2021_24213_MOESM1_ESM.pdf]

Supplementary Information

Single-cell transcriptomic analysis reveals disparate effector differentiation pathways in human T_{reg} compartment

Yuechen Luo[1,#], Changlu Xu[1,#], Bing Wang[1,#], Qing Niu[1,#], Xiuhua Su[1,#], Yingnan Bai[2], Shuxian Zhu[1], Chunxiao Zhao[1], Yunyan Sun[1], Jiali Wang[1], Maolan Liu[1], Xiaolei Sun[1], Ge Song[1], Haidong Cui[3], Xiaoli Chen[4], Huifang Huang[5], Haikun Wang[6], Mingzhe Han[1], Erlie Jiang[1], Lihong Shi[1], Xiaoming Feng[1,5]

Contents:

Supplementary Figures 1-12

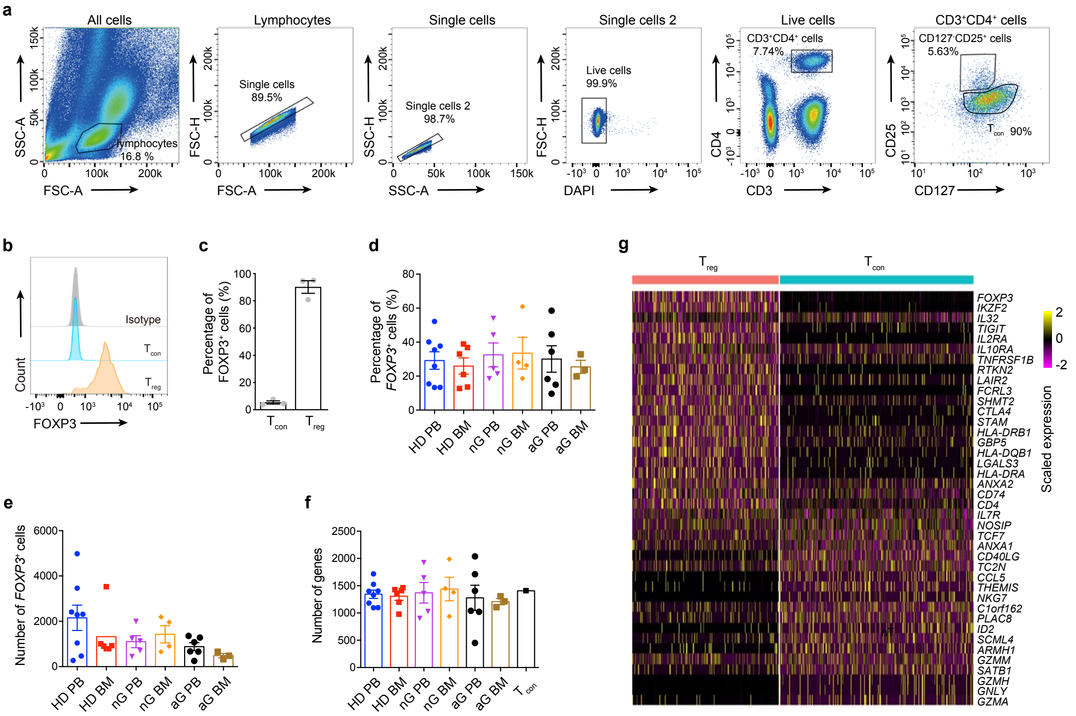

Supplementary Fig. 1. scRNA-seq analysis of T_{reg} and T_{con} cells from healthy donors and allo-HSCT patients. a Enrichment gating strategy of human $DAPI^{-}CD3^{+}CD4^{+}CD25^{+}CD127^{-}$ T_{reg} cells and $DAPI^{-}CD3^{+}CD4^{+}CD25^{-}$ T_{con} cells. **b** The representative *ex vivo* protein staining of FOXP3 in sorted T_{reg} cells and T_{con} cells. **c** The proportion of $FOXP3^{+}$ T_{reg} cells in sorted T_{reg} cells and T_{con} cells ($n = 3$). **d** The percentage of *FOXP3*$^{+}$ cells that were detected by scRNA-seq in sorted T_{reg} cells in each sample (HD PB: $n = 8$, HD BM: $n = 6$, nG PB: $n = 5$, nG BM: $n = 4$, aG PB: $n = 6$, nG BM: $n = 3$). **e** The number of *FOXP3*$^{+}$ cells collected in each sample for subsequent scRNA-seq analyses (HD PB: $n = 8$, HD BM: $n = 6$, nG PB: $n = 5$, nG BM: $n = 4$, aG PB: $n = 6$, nG BM: $n = 3$). **f** The number of genes of *FOXP3*$^{+}$ cells for each sample (HD PB: $n = 8$, HD BM: $n = 6$, nG PB: $n = 5$, nG BM: $n = 4$, aG PB: $n = 6$, nG BM: $n = 3$, T_{con}: $n = 1$). **g** Heatmap showing the expression of the top 20 genes (by fold change) differentially expressed in T_{con} and T_{reg} cells from the HD PB samples. Data are presented as mean values ± SEM. Source data are provided as a Source Data file.

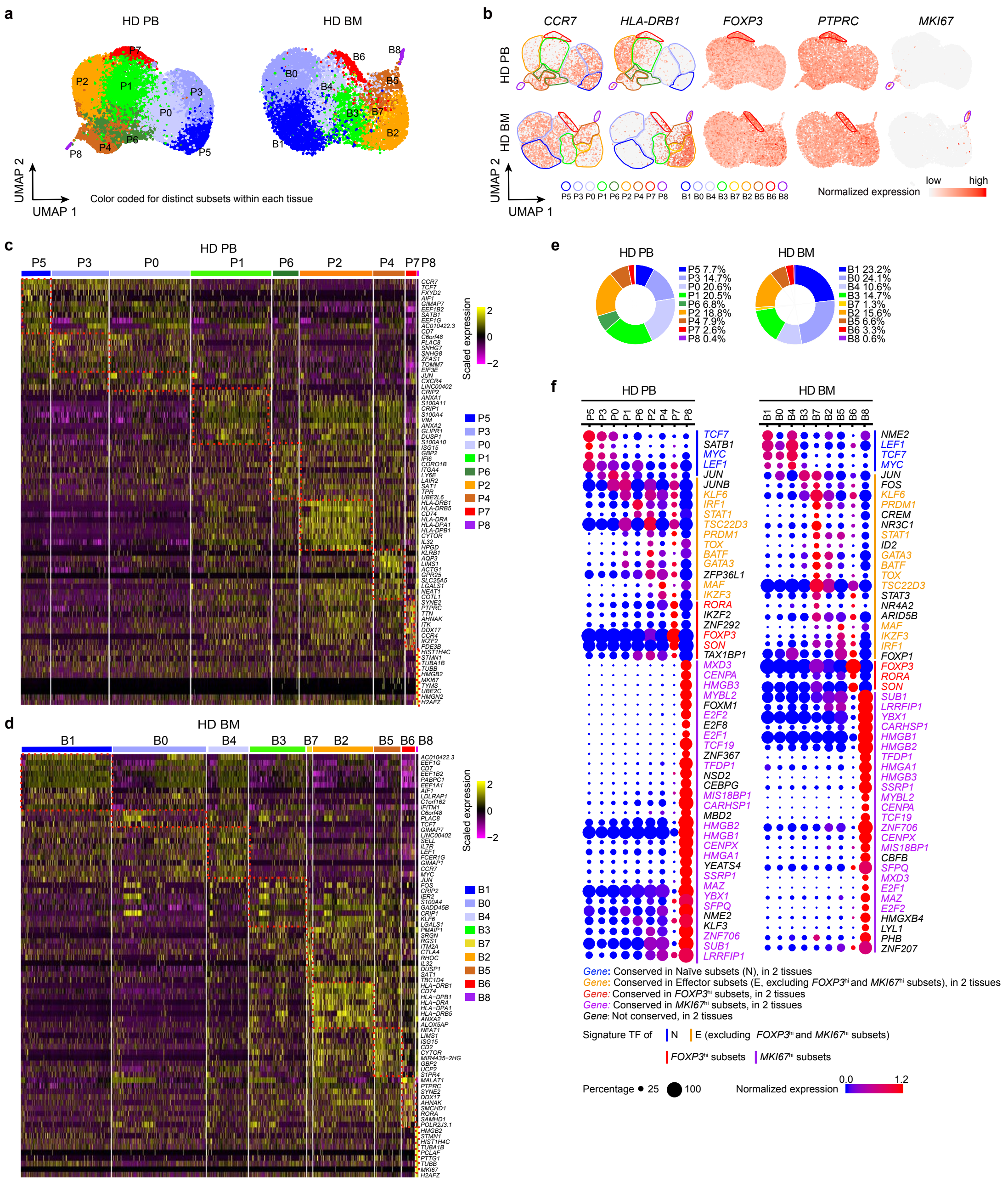

Supplementary Fig. 2. scRNA-seq reveals the heterogeneity of HD PB and BM T_{reg} cells. a UMAP plot of single-cell transcriptomes of T_{reg} cells from HD PB and BM samples, colored by subsets. **b** Projection of *CCR7*, *HLA-DRB1*, *FOXP3*, *PTPRC* and *MKI67* expression onto the UMAP plot. **c,d** Heatmaps showing the expression of the top 10 (by fold change) genes differentially expressed in each HD PB (**c**) and BM (**d**) T_{reg} cell subset, excluding the ribosomal and mitochondrial genes. **e** The proportions of different T_{reg} cell subsets in different HD tissues. **f** Dot plots showing the expression of TFs in marker genes for each T_{reg} cell subset. Source data are provided as a Source Data file.

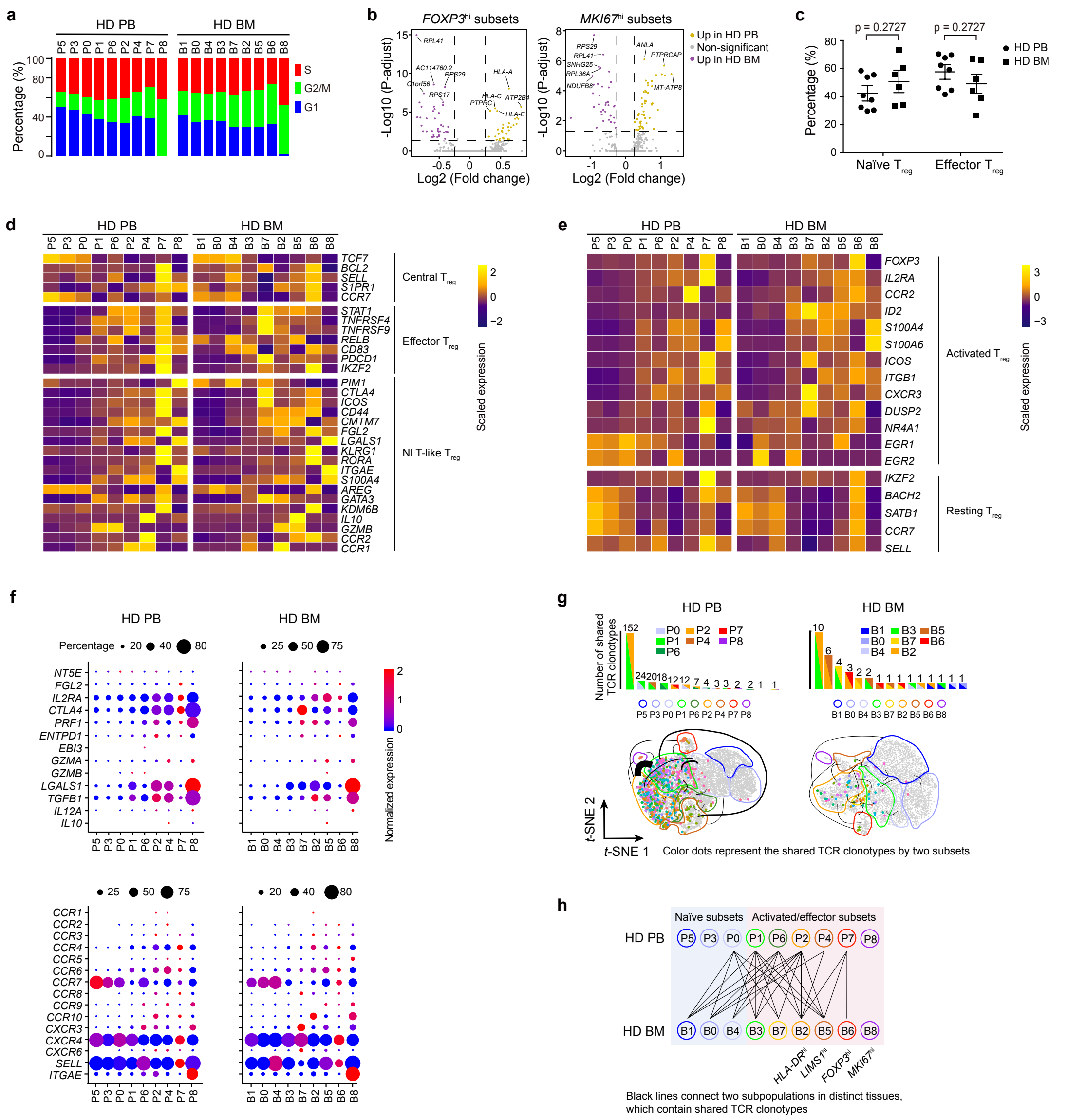

Supplementary Fig. 3. Characterization of HD PB and BM T_{reg} cell subsets. a The proportions of cells in distinct phases of the cell cycle for each subset. **b** Volcano plot shows mRNA expression differences between HD PB-derived *FOXP3*^{hi} subset and HD BM-derived *FOXP3*^{hi} subset, or between HD PB-derived *MKI67*^{hi} subset and HD BM-derived *MKI67*^{hi} subset, based on a cutoff value of 2-fold differential expression and adjusted p < 0.05. **c** The proportions of naïve and effector T_{reg} cell subsets in different HD tissues (HD PB: n = 8, HD BM: n = 6). **d,e** Heatmaps showing the expression of characteristic genes for T_{reg} subsets, which were reported in previous articles (**d**: *Immunity*. 2019 Feb 19;50(2):493-504.e7. doi: 10.1016/j.immuni.2019.01.001. **e**: *Nat Immunol*. 2018 Mar;19(3):291-301. doi: 10.1038/s41590-018-0051-0.). **f** Dot plots showing the expression of suppression (top) and migration (bottom) genes in each subset. **g** The shared TCR distribution of T_{reg} cells between any two subsets, colored by TCR clonotypes. The thickness of the black solid lines representing the relative numbers of shared TCRs. The insert pictures show the numbers of TCRs shared by two subsets. **h** The shared TCR distribution of T_{reg} cells between any two subpopulations from different tissues. The black lines connected two subpopulations, which contained shared TCRs. In **c**, p values determined by two-sided unpaired t-test; data are presented as mean values ± SEM. Source data are provided as a Source Data file.

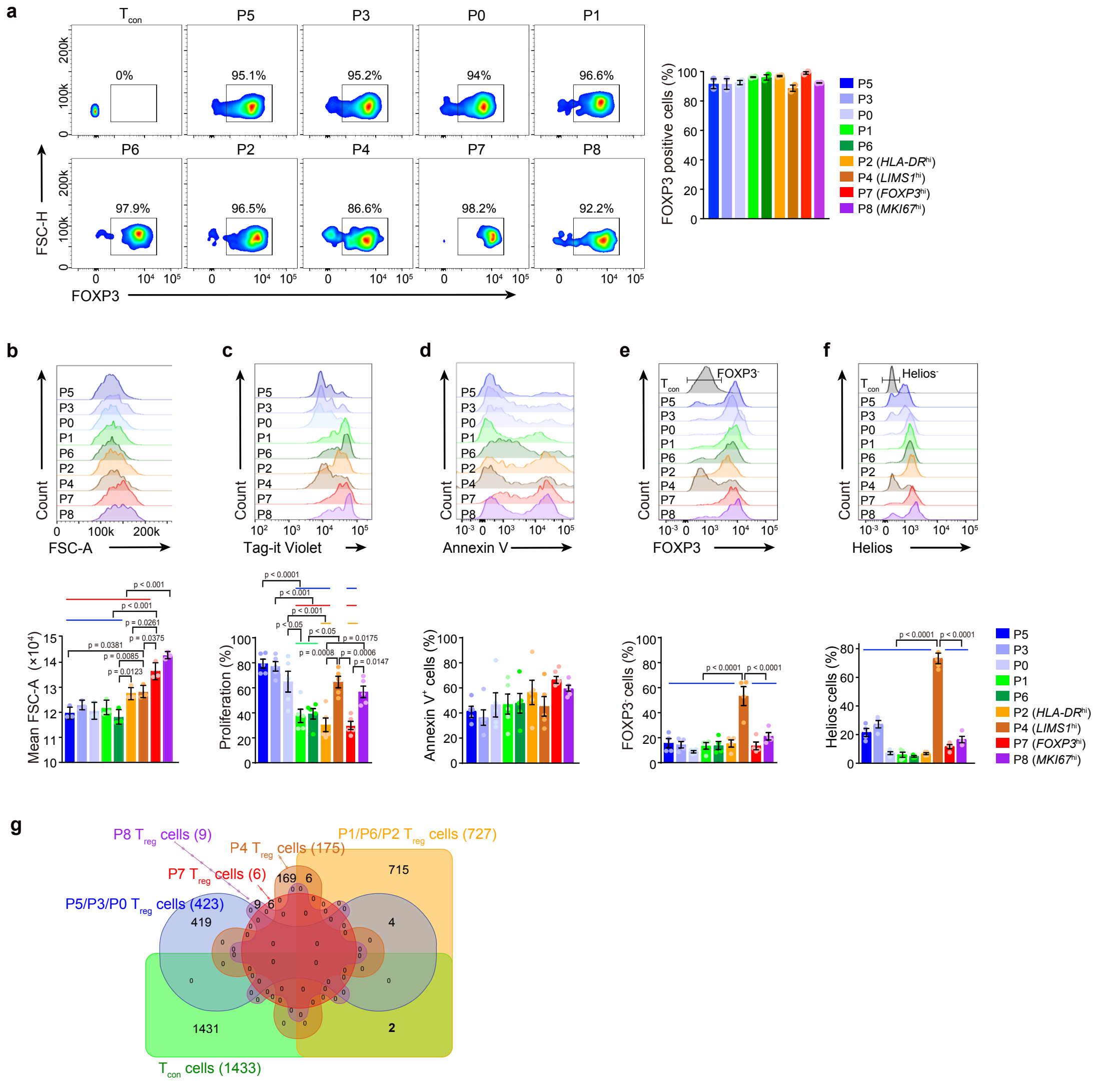

Supplementary Fig. 4. *In vitro* analyses of the characteristics of HD PB T_{reg} cell subsets. a The percentage of FOXP3 positive cells in each T_{reg} cell subset after FACS sorting ($n = 2$). **b** Flow cytometry and its quantification of FSC-A in each HD PB T_{reg} cell subset from PBMC ($n = 3$). Blue line indicated that P7 was compared with P5, P3, P0, P1 or P6; red line indicated that P8 was compared with P5, P3, P0, P1, P6, P2 or P4. **c** The proliferation of Tag-it Violet-labeled different T_{reg} cell subsets, after 96 h *in vitro* culture ($n = 5$). Blue line indicated that P5 was compared with P1, P6, P2 or P7; red line indicated that P3 was compared with P1, P6, P2 or P7; orange line indicated that P0 was compared with P2 or P7; green line indicated that P0 or P4 was compared with P1 or P6. **d** Flow cytometry and its quantification of Annexin V in each HD PB T_{reg} cell subset ($n = 5$). **e** Flow cytometry and its quantification of FOXP3 in each HD PB T_{reg} cell subset, after 96 h *in vitro* culture ($n = 4$). Blue line indicated that P4 was compared with P5, P3, P0, P1, P6, P2, P7 or P8. **f** Flow cytometry and its quantification of Helios in each HD PB T_{reg} cell subset from PBMC ($n = 4$). Blue line indicated that P4 was compared with P5, P3, P0, P1, P6, P2, P7 or P8. **g** The number of shared TCRs between HD PB T_{reg} cell subsets and HD PB T_{con} cells. In **b-f**, experiments were repeated at least three times; p values were determined by one-way ANOVA, part of the statistical significance was shown in the pictures and the whole ANOVA results were given in Supplementary Data 11; data are presented as mean values ± SEM. Source data are provided as a Source Data file.

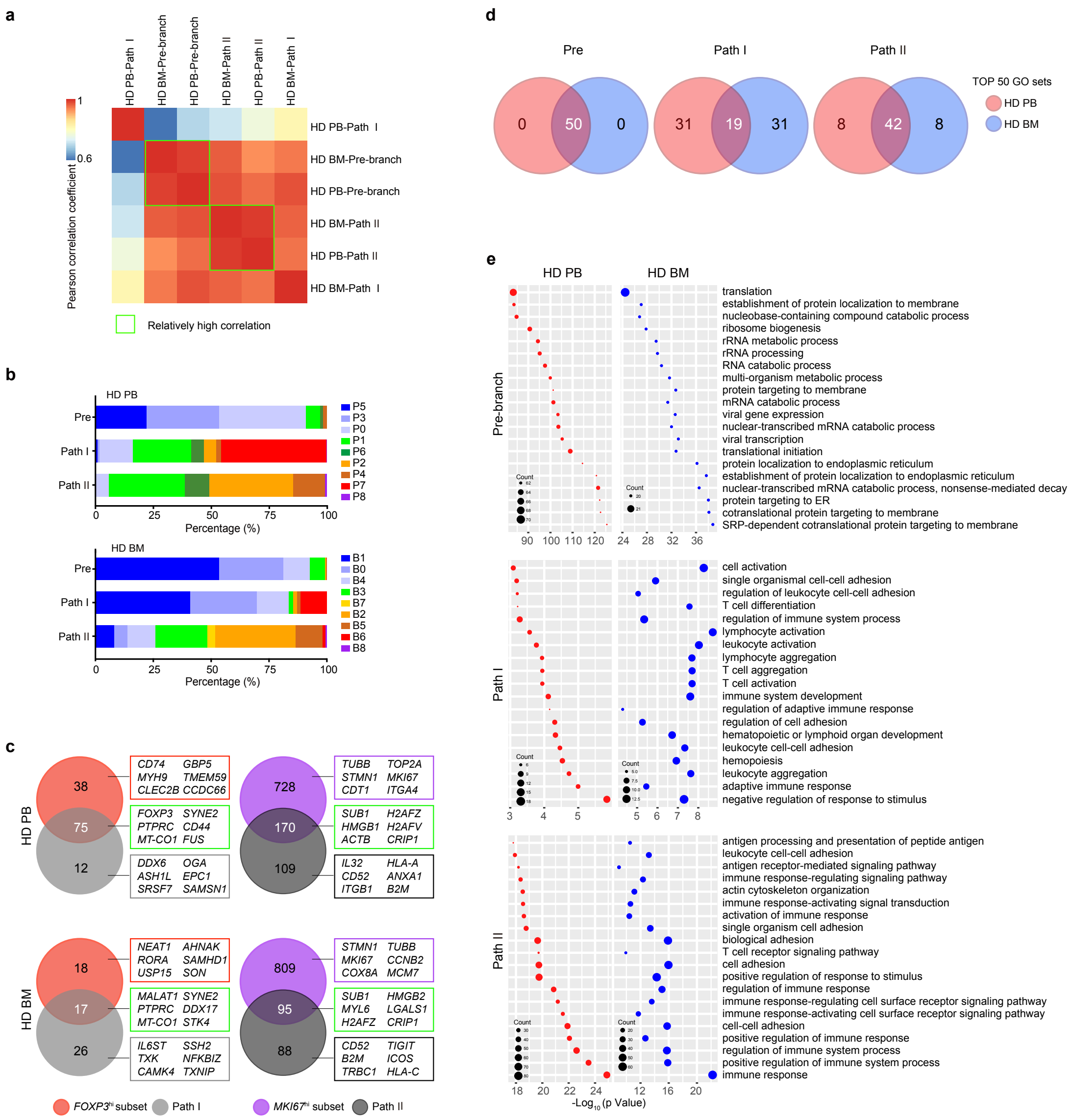

Supplementary Fig. 5. scRNA-seq reveals the heterogeneity of different HD PB and BM T_{reg} Paths. a Correlograms visualizing the correlation of single-cell gene expression profiles between different paths across tissues. **b** The proportions of T_{reg} cell subsets in different Paths. **c** The number of overlapped marker genes between *FOXP3*^{hi} subset and Path I cells, or between *MKI67*^{hi} subset and Path II cells, from HD PB and HD BM. The representative genes were shown in rectangular box. **d** The number of overlapped Gene Ontology terms between T_{reg} cell paths (top 50, by p value). **e** Representative overlapping Gene Ontologies from **d**. The size of each dot represented the number of differentially expressed genes. Source data are provided as a Source Data file.

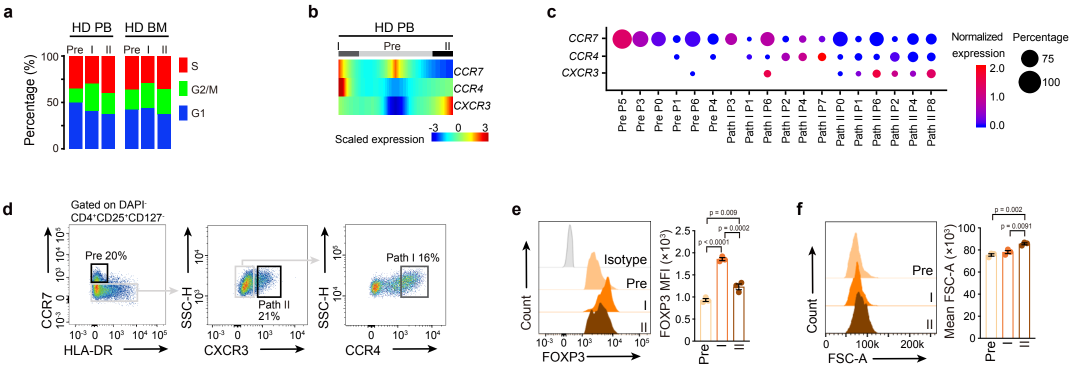

g

Gene	P. adjust	Paths
TCF7	0	Pre
MYC	1.5639×10^{-142}	Pre
SATB1	7.97×10^{-139}	Pre
LEF1	1.128×10^{-130}	Pre
FOXP3	2.926×10^{-206}	Path I
RORA	2.67×10^{-17}	Path I
IKZF2	7.1892×10^{-17}	Path I
ZNF292	1.8598×10^{-10}	Path I
SON	6.9513×10^{-9}	Path I
SUB1	0	Path II
PRDM1	5.143×10^{-285}	Path II
LRRFIP1	2.238×10^{-209}	Path II
TOX	8.321×10^{-202}	Path II
BATF	3.356×10^{-178}	Path II
GATA3	5.455×10^{-169}	Path II
HMGB1	2.265×10^{-164}	Path II
KLF6	1.201×10^{-161}	Path II
TSC22D3	2.855×10^{-129}	Path II
HMGB2	7.892×10^{-117}	Path II
STAT1	1.3×10^{-107}	Path II

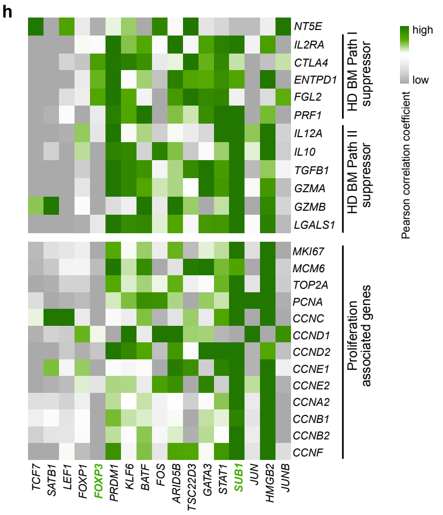

Supplementary Fig. 6. The characteristics of different T_{reg} Paths. a The proportions of cells in distinct phases of the cell cycle for each path. **b** Pseudotemporal gene-expression profiles of the indicated surface genes for each path. **c** Dot plots showing the expression of the indicated surface genes for different HD PB T_{reg} cell cluster-path subpopulations. **d** Gating strategy for HD PB T_{reg} cell path sorting. **e, f** Flow cytometry and its quantification of FOXP3 or FSC-A in each HD PB T_{reg} cell Path ($n = 3$). **g** The p values of TFs from each HD PB Path marker genes. **h** Correlograms visualizing the correlation of single-cell gene expression profiles between TFs and suppression or proliferation genes in HD BM samples. In **e**, **f**, experiments were repeated three times; p values were determined by one-way ANOVA; data are presented as mean values ± SEM. Source data are provided as a Source Data file.

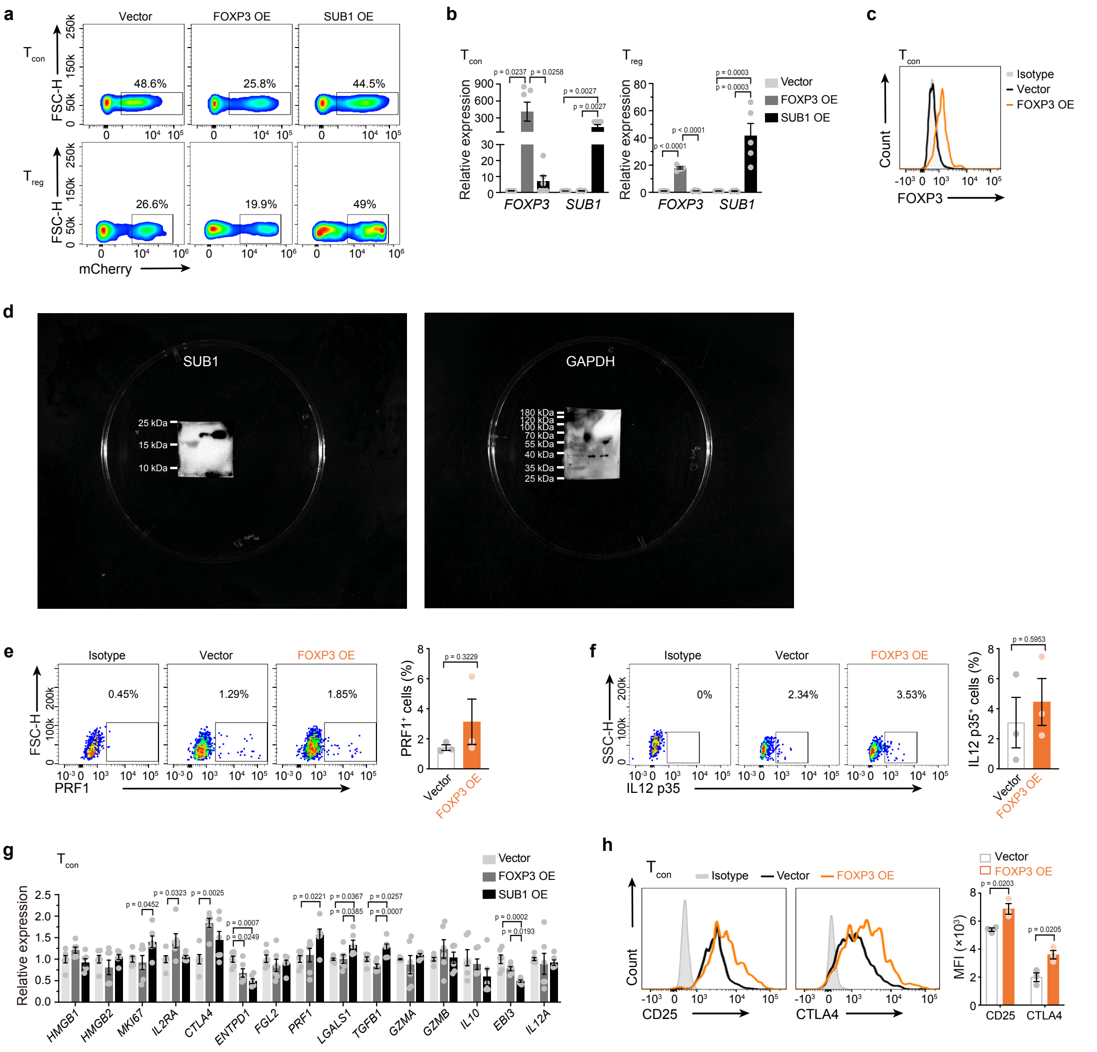

Supplementary Fig. 7. Transcription factor basis for T_{reg} cell differentiation Pathway. a The virus infection efficiency of T_{con} or T_{reg} cells, detected by flow cytometry. Experiment repeated at least three times. **b** *FOXP3* and *SUB1* mRNA levels in HD PB T_{con} ($n = 6$) or T_{reg} ($n = 5$) cells, 5 days after transfection with FOXP3 or SUB1 overexpression virus, as measured by RT-qPCR. Experiment repeated at least three times. **c** FOXP3 protein levels in HD PB T_{con} cells, 5 days after transfection with FOXP3 overexpression virus, as measured by flow cytometry. Experiment repeated at least three times. **d,** Uncropped scans of western blots (Fig. 5**d**). **e,f** Expression of PRF1 and IL12 p35 in HD PB T_{reg} cells, 5-6 days after transfection with FOXP3 overexpression virus, as measured by flow cytometry ($n = 3$). Experiment repeated three times. **g** Expression of the mRNA level of indicated genes in HD PB T_{con} cells, 5 days after transfection with FOXP3 or SUB1 overexpression virus, as measured by RT-qPCR ($n = 6$). Experiment repeated at least three times. **h** Expression of CD25 and CTLA4 in HD PB T_{con} cells, 5 days after transfection with FOXP3 overexpression virus, as measured by flow cytometry ($n = 3$). Experiment repeated three times. In **b**, **g**, p values were determined by One-way ANOVA; In **e**, **f**, **h**, p values determined by two-sided unpaired *t*-test. Data are presented as mean values ± SEM. Source data are provided as a Source Data file.

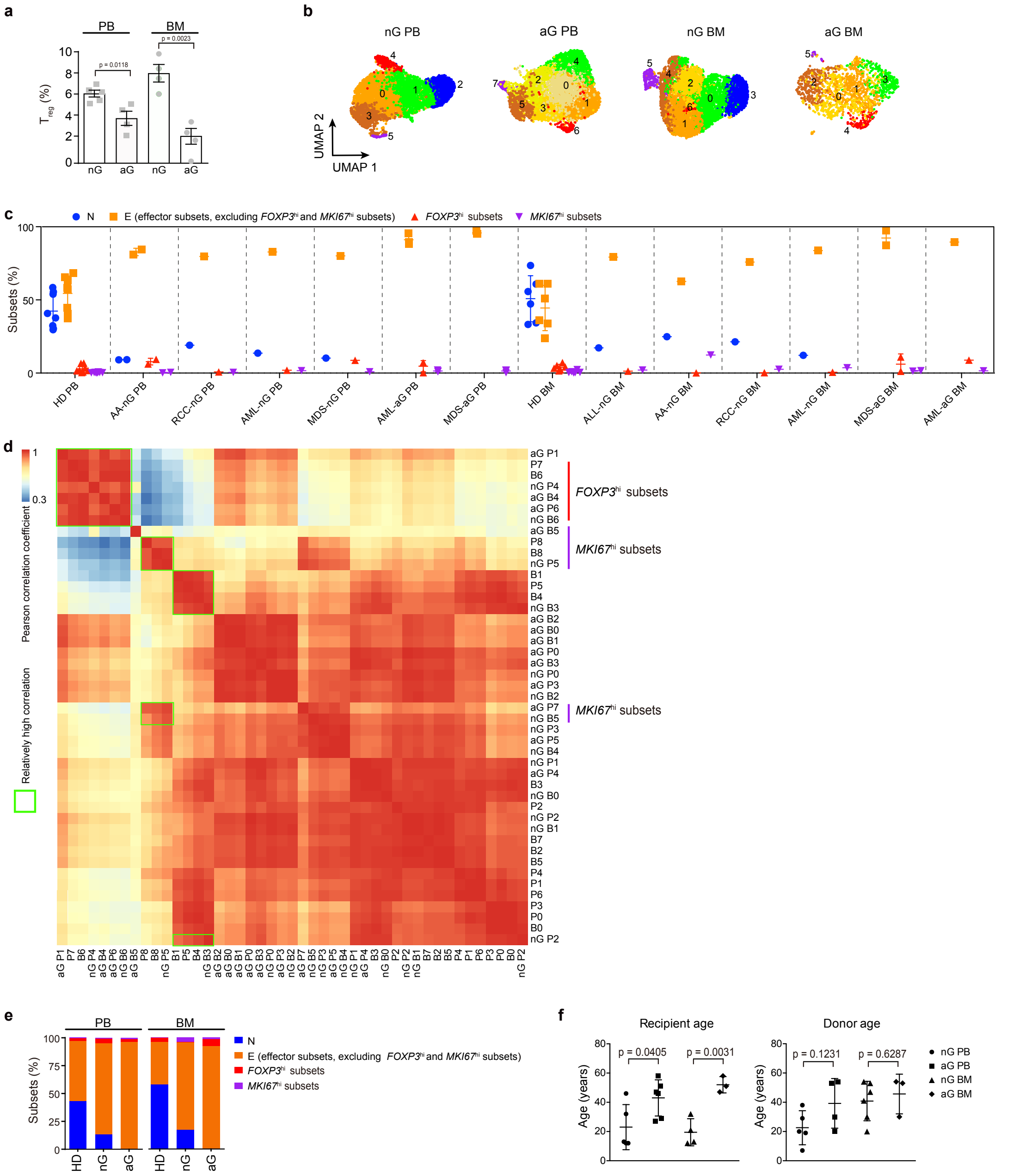

Supplementary Fig. 8. T$_{reg}$ cell subset dysfunction in allo-HSCT patients with or without aGVHD. a The frequencies of T$_{reg}$ cells in the PB and BM from allo-HSCT patients (nG PB: *n* = 5, aG PB: *n* = 4, nG BM: *n* = 4, nG BM: *n* = 4). **b** UMAP plot of single-cell transcriptomes of T$_{reg}$ cells from allo-HSCT patients, colored by subsets. **c** The percentage of certain subsets in healthy donors and patients cross different primary diseases (HD PB: *n* = 8, AA-nG PB: *n* = 2, RCC-nG PB: *n* = 1, AML-nG PB: *n* = 1, MDS-nG PB: *n* = 1, AML-aG PB: *n* = 3, MDS-aG PB: *n* = 3, HD BM: *n* = 6, ALL-nG BM: *n* = 1, AA-nG BM: *n* = 1, RCC-nG BM: *n* = 1, AML-nG BM: *n* = 1, MDS-aG BM: *n* = 2, AML-aG BM: *n* = 1). **d** Correlograms visualizing the correlation of single-cell gene expression profiles between subsets in PB and BM samples from healthy donors, non-aGVHD and aGVHD patients. **e** The proportion of certain subsets in PB and BM samples from healthy donors, non-aGVHD and aGVHD patients. **f** The recipient and donor age of allo-HSCT patients (nG PB: *n* = 5, nG BM: *n* = 4, aG PB: *n* = 6, nG BM: *n* = 3). In **a**,**f**, p values determined by two-sided unpaired *t*-test. Data are presented as mean values ± SEM. Source data are provided as a Source Data file.

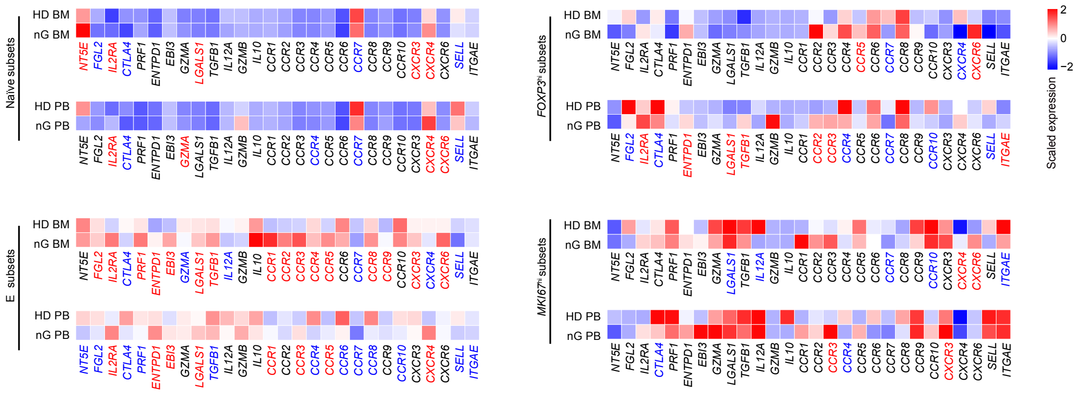

Supplementary Fig. 9. The dynamic changes of migration and suppression-associated genes in different T_{reg} cell subsets from non-aGVHD patients. Heatmaps showing the expression of migration and suppression-associated genes in different T_{reg} cell subsets. The blue font indicated that the genes were significantly down-regulated in non-aGVHD patients, compared with healthy donors ($p < 0.05$). The red font indicated that the genes were significantly up-regulated in non-aGVHD patients, compared with healthy donors ($p < 0.05$). Gene normalized expressions were used and p values were determined by Wilcoxon rank-sum test.

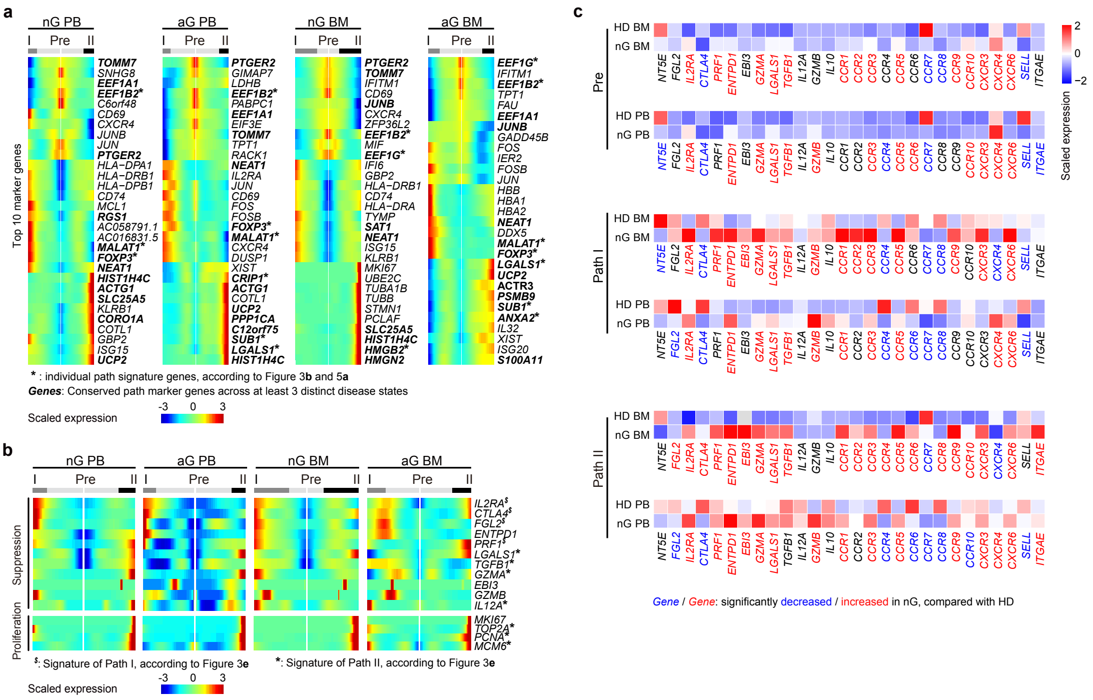

Supplementary Fig. 10. T_{reg} cell Path dysfunction in allo-HSCT patients with or without aGVHD. a Pseudotemporal gene-expression profiles of the top 10 (by fold change) marker genes for each path, excluding the ribosomal and mitochondrial genes. **b** Pseudotemporal gene-expression profiles of suppression and proliferation-associated genes in the T_{reg} cell paths. **c** Heatmaps showing the expression of migration and suppression-associated genes in different Path cells. The blue font indicated that the genes were significantly down-regulated in non-aGVHD patients, compared with healthy donors ($p < 0.05$). The red font indicated that the genes were significantly up-regulated in non-aGVHD patients, compared with healthy donors ($p < 0.05$). In **c**, gene normalized expressions were used and p values were determined by Wilcoxon rank-sum test.

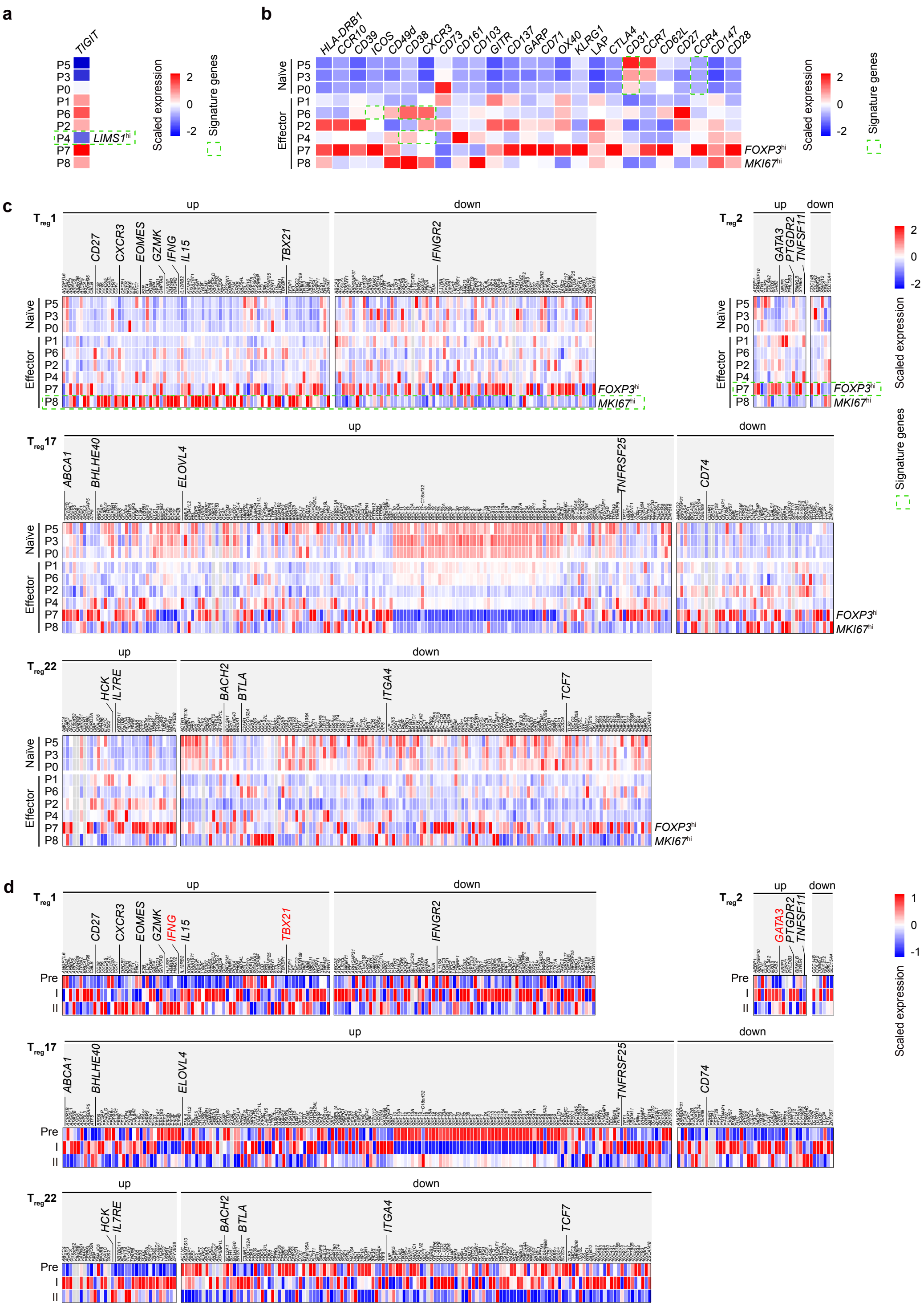

Supplementary Fig. 11. The expression of signature genes in T_{reg} cells. a Heatmap showing the expression of *TIGIT* in HD PB T_{reg} cell subsets. **b** Heatmaps showing the expression of indicated genes in HD PB T_{reg} cell subsets. The indicated genes were reported in a previous article (*J Immunol*. 2015 Sep 1;195(5):2030-7. doi: 10.4049/jimmunol.1500703). **c, d** Heatmaps showing the expression of Th-like characteristic genes in T_{reg} cell subsets (**c**) and T_{reg} cell Paths (**d**). The Th-like characteristic genes were reported in a previous article (*Immunohorizons*. 2020 Oct 9;4(10):585-596. doi:10.4049/immunohorizons.2000037).

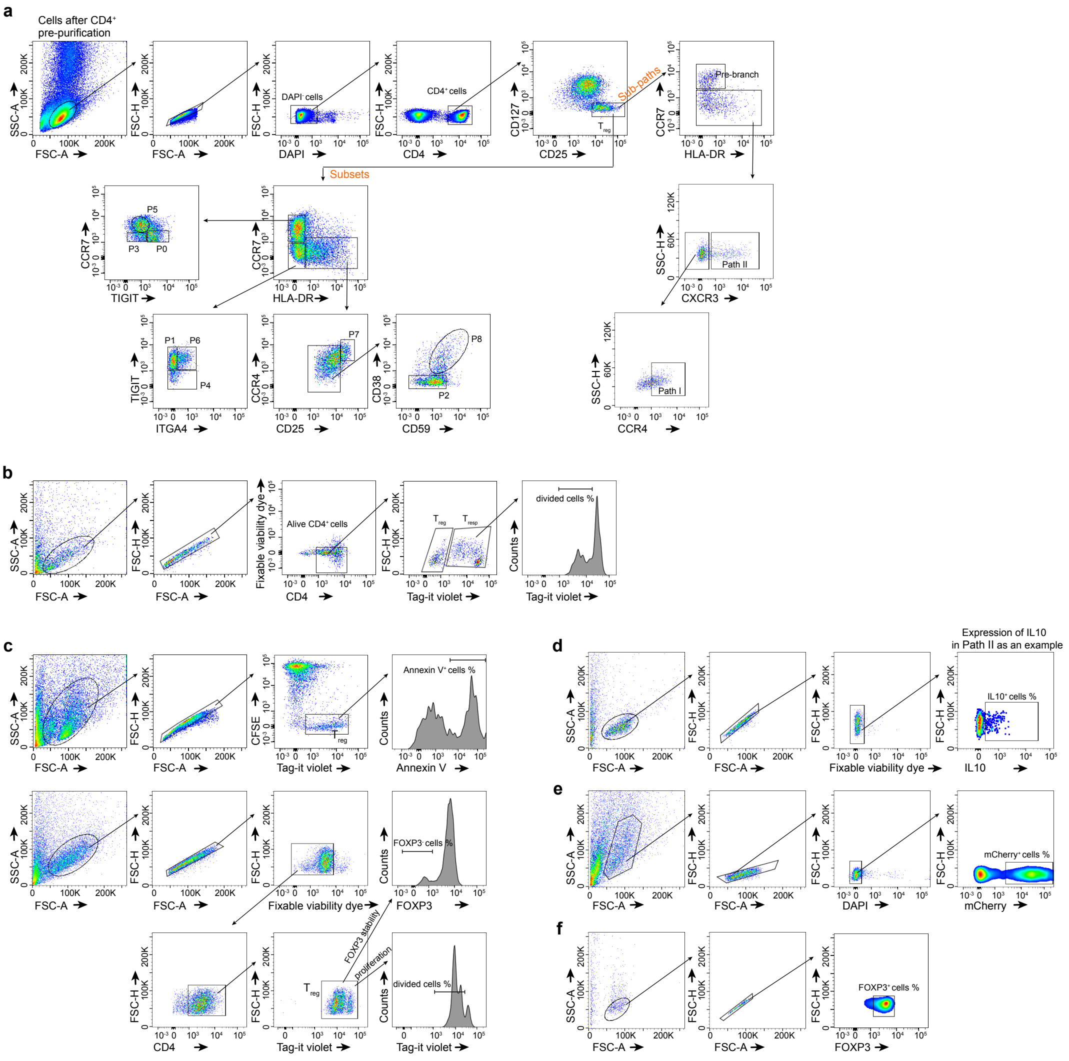

Supplementary Fig. 12. Gating strategies used for cell sorting or downstream analyses. a Gating strategy of T_{reg} cell (DAPI$^-$CD4$^+$CD25$^+$CD127^{low}), T_{reg} subsets or sub-path cells from healthy donor's PBMC for cell sorting presented on Fig. 2**b**, Supplementary Fig. 6**d** , or for various *in vitro* assays presented on Fig. 2**e**, 4**b**, 4**d**, 4**e** Supplementary Fig. 4**c-e** or characterizations of the subsets or sub-path cells presented on Fig. 2**c**, **d**, Supplementary Fig. 4**b**, **f**. and Fig. 4**a**, **c**, Supplementary Fig. 6**e**, **f**. **b** Gating strategy to analysis *in vitro* suppression ability of subsets or sub-path cells presented on Fig. 2**e**, 4**e**. **c** Gating strategy of Tag-it violet labeled T_{reg} cells to characterize the indicated features (top: apoptosis, bottom: stability and proliferation) of *in vitro* cultured subsets and sub-path cells (Supplementary Fig. 4**c-e**, Fig. 4**d**). **d** Gating strategy of single alive cells to determine the expression of indicated proteins in sorted sub-path cells or FOXP3- or SUB1-overexpressed cells (Fig. 4**b**, Fig. 5**c**, **f**, **g**, **i**, **j**, Supplementary Fig. 7**c**, **e**, **f, h**). **e** Gating strategy to sort the FOXP3- or SUB1-overexpressed T_{reg} (Supplementary Fig. 7**a**-bottom, Fig. 5**c-j**, Supplementary Fig. 7**d-f**) and T_{con} cells (Supplementary Fig. 7**a**-top, 7**b**, **c**, **g**, **h**) for downstream analysis. **f** Gating strategy to determine the purity of sorted subset cells (Supplementary Fig. 4**a**).
