# Supplementary material for: Single-cell transcriptomic analysis reveals disparate effector differentiation pathways in human Treg compartment
Source: Nat Commun. 2021 Jun 23;12:3913. doi: 10.1038/s41467-021-24213-6 (PMC8222404; doi:10.1038/s41467-021-24213-6)
Supplement: Supplementary file 2 — Description of Additional Supplementary Files [file 41467_2021_24213_MOESM2_ESM.docx]

**Description of Additional Supplementary Files**

Title: Supplementary Data 1.

Description: Characteristics of healthy donors, non-aGVHD and aGVHD patients.

Title: Supplementary Data 2.

Description: Sequencing information of individual samples.

Title: Supplementary Data 3.

Description: Gene signatures discriminating Treg cells from Tcon cells.

Title: Supplementary Data 4.

Description: Gene signatures of Treg cell clusters.

Title: Supplementary Data 5.

Description: The RNA expression differences between HD PB FOXP3hi subset and HD BM FOXP3hi subset or between HD PB MKI67hi subset and HD BM MKI67hi subset.

Title: Supplementary Data 6.

Description: List of gene sets related to specific Treg cell features.

Title: Supplementary Data 7.

Description: TCR clonotypes of single Treg and Tcon cells.

Title: Supplementary Data 8.

Description: Gene signatures of Treg cell differentiation paths.

Title: Supplementary Data 9.

Description: GO analysis of HD PB and BM and Treg cell differentiation paths.

Title: Supplementary Data 10.

Description: Information of reagents and primers.

Title: Supplementary Data 11.

Description: The one-way ANOVA results.
