# Supplementary material for: Single-cell transcriptomic analysis reveals disparate effector differentiation pathways in human Treg compartment
Source: Nat Commun. 2021 Jun 23;12:3913. doi: 10.1038/s41467-021-24213-6 (PMC8222404; doi:10.1038/s41467-021-24213-6)
Supplement: Supplementary file 4 — Reporting Summary [file 41467_2021_24213_MOESM4_ESM.pdf]

nature research

Corresponding author(s): Xiaoming Feng, Lihong Shi, Erlie Jiang, Mingzhe Han

Reporting Summary

Nature Research wishes to improve the reproducibility of the work that we publish. This form provides structure for consistency and transparency in reporting. For further information on Nature Research policies, see Authors & Referees and the Editorial Policy Checklist.

Statistical parameters

When statistical analyses are reported, confirm that the following items are present in the relevant location (e.g. figure legend, table legend, main text, or Methods section).

n/a	Confirmed	
☐	☒	The exact sample size (*n*) for each experimental group/condition, given as a discrete number and unit of measurement
☐	☒	An indication of whether measurements were taken from distinct samples or whether the same sample was measured repeatedly
☐	☒	The statistical test(s) used AND whether they are one- or two-sided<br>*Only common tests should be described solely by name; describe more complex techniques in the Methods section.*
☐	☒	A description of all covariates tested
☐	☒	A description of any assumptions or corrections, such as tests of normality and adjustment for multiple comparisons
☐	☒	A full description of the statistics including central tendency (e.g. means) or other basic estimates (e.g. regression coefficient) AND variation (e.g. standard deviation) or associated estimates of uncertainty (e.g. confidence intervals)
☐	☒	For null hypothesis testing, the test statistic (e.g. *F*, *t*, *r*) with confidence intervals, effect sizes, degrees of freedom and *P* value noted<br>*Give P values as exact values whenever suitable.*
☒	☐	For Bayesian analysis, information on the choice of priors and Markov chain Monte Carlo settings
☒	☐	For hierarchical and complex designs, identification of the appropriate level for tests and full reporting of outcomes
☐	☒	Estimates of effect sizes (e.g. Cohen's *d*, Pearson's *r*), indicating how they were calculated
☐	☒	Clearly defined error bars<br>*State explicitly what error bars represent (e.g. SD, SE, CI)*

Our web collection on statistics for biologists may be useful.

Software and code

Policy information about availability of computer code

Data collection	The FlowJo (version 7.6.1), R (version 3.6) were included for data collection in this research.
Data analysis	The FlowJo (version 7.6.1), GraphPad Prism (version 8.3.0.538), R package Seurat (version 3.0.2), CellRanger (version 3.0.2), Monocle (version 2.12.0), Bioconductor package GSVA (version 1.16.0) and DAVID (https://david-d.ncifcrf.gov, version 6.8) were included for data analysis in this research.

For manuscripts utilizing custom algorithms or software that are central to the research but not yet described in published literature, software must be made available to editors/reviewers upon request. We strongly encourage code deposition in a community repository (e.g. GitHub). See the Nature Research guidelines for submitting code & software for further information.

Data

Policy information about availability of data

All manuscripts must include a data availability statement. This statement should provide the following information, where applicable:

- Accession codes, unique identifiers, or web links for publicly available datasets
- A list of figures that have associated raw data
- A description of any restrictions on data availability

The scRNA-seq data and scTCR-seq data sets have been deposited in the Gene Expression Omnibus (GEO) at GSE175604. Date usage shall be in full compliance with

the Regulations on Management of Human Genetic Resources in China. All other relevant data supporting the key findings of this study are available within the article and its Supplementary Information files or from the corresponding author upon reasonable request. Source data are provided with this paper. A reporting summary for this Article is available as a Supplementary Information file.

Field-specific reporting

Please select the best fit for your research. If you are not sure, read the appropriate sections before making your selection.

- [x] Life sciences
- [ ] Behavioural & social sciences
- [ ] Ecological, evolutionary & environmental sciences

For a reference copy of the document with all sections, see nature.com/authors/policies/ReportingSummary-flat.pdf

Life sciences study design

All studies must disclose on these points even when the disclosure is negative.

Sample size	Sample size for scRNA-seq and scTCR-seq were determined by the availability of patient samples. No statistical tests were performed for sample size calculation. The exact number of samples used for each figure is informed in each legend or manuscript description.
Data exclusions	No data was excluded.
Replication	Each experiment in this study was independently repeated for at least 3 times to generate final conclusion. The replication of experiments showed similar results or trend.
Randomization	All the human specimens and organisms involved in this study were allocated in randomization.
Blinding	Blinding was performed in data collection stage of scRNA-seq and TCR-seq, and the cellular and biochemical experiments were not performed in blind, since analysis were performed under selectively grouping and conditions.

Reporting for specific materials, systems and methods

Materials & experimental systems

n/a	Involved in the study	
x		Unique biological materials
	x	Antibodies
	x	Eukaryotic cell lines
x		Palaeontology
x		Animals and other organisms
	x	Human research participants

Methods

n/a	Involved in the study	
x		ChIP-seq
	x	Flow cytometry
x		MRI-based neuroimaging

Antibodies

Antibodies used

For flow cytometry:
anti-human CD4 PE-Cyanine7 357410 A161A1 Biolegend 1/200
anti-human CD4 BV785 300553 RPA-T4 Biolegend 1/200
anti-human CD3 APC-Cyanine7 317341 OKT3 Biolegend 1/200
anti-human CD25 APC 302610 BC96 Biolegend 1/100
anti-human CD25 BV711 356137 M-A251 Biolegend 1/100
anti-human CD127 FITC 351312 A019D5 Biolegend 1/100
anti-human CD127 BV510 351331 A019D5 Biolegend 1/100
anti-human CCR7 (CD197) BV650 353234 G043H7 Biolegend 1/100
anti-human CCR7 (CD197) APC 353213 G043H7 Biolegend 1/100
anti-human HLA-DR BV605 307639 L243 Biolegend 1/100
anti-human TIGIT APC 372706 A15153G Biolegend 1/50
anti-human ITGA4 (CD49D) APC-Cyanine7 304328 9F10 Biolegend 1/50
anti-human CCR4 (CD194) PerCP-Cyanine5.5 359405 L291H4 Biolegend 1/100
anti-human CCR4 (CD194) APC 359407 L291H4 Biolegend 1/100
anti-human CD38 PE-Cyanine7 356608 HB-7 Biolegend 1/100
anti-human CD59 FITC 304706 H19 Biolegend 1/100

anti-human CD59 PE 304707 H19 eBioscience 1/100
anti-human CXCR3 (CD183) PE 2009783 CEW33D Invitrogen 1/100
anti-human CXCR3 (CD183) FITC 353704 G025H7 Biolegend 1/50
anti-human CD161 (KLRB1) PE/Cy7 339917 HP-3G10 Biolegend 1/100
anti-human FOXP3 PE 320107 206D Biolegend 1/100
anti-human FOXP3 PE 4331087 236A/E7 eBioscience 1/50
anti-human Helios (IKZF2) FITC 137204 22F6 Biolegend 1/100
anti-human CD152 (CTLA-4) PE 369603 BNI3 Biolegend 1/100
anti-human CD152 (CTLA-4) PE-Cyanine7 369613 BNI3 Biolegend 1/100
anti-human IL-10 PerCP/Cyanine5.5 501417 JES3-9D7 Biolegend 1/50
anti-Human TGF-β1 BV421 562962 TW4-9E7 BD Bioscience 1/50
anti-human Granzyme A PE-Cyanine7 25-9177-41 CB9 eBioscience 1/100
anti-human Granzyme B FITC 515403 GB11 Biolegend 1/100
anti-human LAP (TGF-β1) APC 349705 TW4-6H10 Biolegend 1/100
anti-human Perforin BV510 308119 dG9 Biolegend 1/50
anti-human IL12 p35 eFluor® 660 50-7359-41 2247466 Invitrogen 1/50
anti-human KI67 BV421 562899 Clone B56 BD Bioscience 1/100
Mouse IgG1, κ Isotype Ctrl Antibody APC 400119 MOPC-21 Biolegend 1/200
Mouse IgG1, κ Isotype Ctrl Antibody APC-Cyanine7 400127 MOPC-21 Biolegend 1/200
Mouse IgG1, κ Isotype Ctrl Antibody PE 400111 MOPC-21 Biolegend 1/100
Mouse IgG1, κ Isotype Ctrl Antibody PE/Cy7 400125 MOPC-21 Biolegend 1/200
Mouse IgG1, κ Isotype Ctrl Antibody PerCP/Cyanine5.5 400149 MOPC-21 Biolegend 1/200
Mouse IgG1, κ Isotype Ctrl Antibody FITC 400107 MOPC-21 Biolegend 1/100
Mouse IgG1, κ Isotype Ctrl Antibody BV421 400157 MOPC-21 Biolegend 1/200

For western blot:
anti-SUB1 antibody rabbit pAb HPA001311 Sigma-Aldrich 1/3000
anti-GAPDH antibody rabbit mAb D16H11 Cell Signaling Technology 1/3000

Validation

All antibodies used in this study were obtained from commercial source, and validated according to manufacturers' instruction.

Eukaryotic cell lines

Policy information about cell lines

Cell line source(s)

293T

Authentication

The 293T cell line was originally obtained from the American Type Culture Collection (ATCC).

Mycoplasma contamination

The 293T cell line was tested negative for mycoplasma contamination.

Commonly misidentified lines
(See ICLAC register)

No commonly misidentified line was involved in this study.

Human research participants

Policy information about studies involving human research participants

Population characteristics

Nine HDs, six non-aGVHD patients and six aGVHD patients were enrolled in this study. aGVHD was staged according to modified Glucksberg criteria. Their ages ranged from 12 to 58, with a median age of 32. All HSCT patients received aGVHD prophylaxis with tacrolimus or ciclosporin plus short-term methylprednisolone, with or without ruxolitinib.

Recruitment

The inclusion criteria of patients were allogeneic stem cell transplantation, donor cell chimerism of CD3+ cells > 95%, and no active infections (i.e., cytomegalovirus or hepatitis B virus). This study was approved by the Ethics Committee of the State Key Laboratory of Experimental Hematology, Institute of Hematology and Hospital of Blood Disease, Chinese Academy of Medical Sciences & Peking Union Medical College, Tianjin, China. All the people in this study provided written informed consent for sample collection and data analyses.

Flow Cytometry

Plots

Confirm that:

- [x] The axis labels state the marker and fluorochrome used (e.g. CD4-FITC).
- [x] The axis scales are clearly visible. Include numbers along axes only for bottom left plot of group (a 'group' is an analysis of identical markers).
- [x] All plots are contour plots with outliers or pseudocolor plots.
- [x] A numerical value for number of cells or percentage (with statistics) is provided.

Methodology

Sample preparation	The sample preparation procedure for FC was provided in the Specimen preparation of single-cell suspensions, at Materials and Methods section.
Instrument	Flow cytometry data were acquired on LSR II, FACS Canto II or FACS Aria III (BD Biosciences).
Software	The FlowJo software was used in FC data analysis.
Cell population abundance	The population abundance of Treg cell subsets were provided in Figure 2b, the population abundance of Treg and Tcon cells in PBMC of human recipients were provided in Supplementary figure 1a, and the population abundance of Treg cell paths were provided in Supplementary figure 6d.
Gating strategy	The gating strategy for Treg cell subsets were provided in Figure 2b, the gating strategy for Treg and Tcon cells purification were provided in Supplementary figure 1a, and the gating strategy for Treg cell paths were provided in Supplementary figure 6d. Supplementary figure 12 has been provided as the summery for all FACS gating strategy in this study.

- [x] Tick this box to confirm that a figure exemplifying the gating strategy is provided in the Supplementary Information.
